# Transcript Dynamics at Early Stages of Molecular Interactions of MYMIV with Resistant and Susceptible Genotypes of the Leguminous Host, *Vigna mungo*

**DOI:** 10.1371/journal.pone.0124687

**Published:** 2015-04-17

**Authors:** Anirban Kundu, Anju Patel, Sujay Paul, Amita Pal

**Affiliations:** 1 Division of Plant Biology, Bose Institute, Kolkata 700054, West Bengal, India; 2 Laboratorio de Micología y Biotecnología, Universidad Nacional Agraria, La Molina, Lima, Peru; Gyeongnam National University of Science and Technology, KOREA, REPUBLIC OF

## Abstract

Initial phases of the MYMIV- *Vigna mungo* interaction is crucial in determining the infection phenotype upon challenging with the virus. During incompatible interaction, the plant deploys multiple stratagems that include extensive transcriptional alterations defying the virulence factors of the pathogen. Such molecular events are not frequently addressed by genomic tools. In order to obtain a critical insight to unravel how *V*. *mungo* respond to Mungbean yellow mosaic India virus (MYMIV), we have employed the PCR based suppression subtractive hybridization technique to identify genes that exhibit altered expressions. Dynamics of 345 candidate genes are illustrated that differentially expressed either in compatible or incompatible reactions and their possible biological and cellular functions are predicted. The MYMIV-induced physiological aspects of the resistant host include reactive oxygen species generation, induction of Ca^2+^ mediated signaling, enhanced expression of transcripts involved in phenylpropanoid and ubiquitin-proteasomal pathways; all these together confer resistance against the invader. Elicitation of genes implicated in salicylic acid (SA) pathway suggests that immune response is under the regulation of SA signaling. A significant fraction of modulated transcripts are of unknown function indicating participation of novel candidate genes in restricting this viral pathogen. Susceptibility on the other hand, as exhibited by *V*. *mungo* Cv. T9 is perhaps due to the poor execution of these transcript modulation exhibiting remarkable repression of photosynthesis related genes resulting in chlorosis of leaves followed by penalty in crop yield. Thus, the present findings revealed an insight on the molecular warfare during host-virus interaction suggesting plausible signaling mechanisms and key biochemical pathways overriding MYMIV invasion in resistant genotype of *V*. *mungo*. In addition to inflate the existing knowledge base, the genomic resources identified in this orphan crop would be useful for integrating MYMIV-tolerance trait in susceptible cultivars of *V*. *mungo*.

## Introduction

Plant viral pathogens exhibit an obligate intracellular mode of parasitism that manipulates host resources for their own survival and proliferation [1]. Due to their limited genetic capability, these pathogens entirely depend on the host’s machinery to complete their life cycle. In doing so, they utilize diverse stratagems to conquer the host, upsetting hosts immune response by escaping its surveillance [2, 3]. Disease manifestation is therefore an organized consequence of pathogen establishment and emergence of disease symptoms in the compatible host. In contrast, incompatible response is a different phenomenon altogether. Here, resistant hosts are equipped with a repertoire of specialized armours that recruits a series of coordinated cellular processes to counter infection by impairing the pathogenic effectors. Subsequently, a cascade of signaling events are elicited that culminates with the formulation of appropriate responses enabling the host to defend against the potential invader.

The key aspect in mounting up an effective response relies on the timely perception of the intruder [4, 5]. In plants, this process is unique and achieved by recognition of pathogen-associated molecular patterns (PAMPs) by the host encoded pattern recognition receptors [6, 7]. As a consequence multitude of reactions are initiated including rapid ion fluxes, generation of reactive oxygen species (ROS), reinforcement of cell walls, transcriptional activation of pathogenesis-related (PR) genes and synthesis of antimicrobials [8]. Depending on the type of interaction, signal molecules like salicylic acid (SA), jasmonic acid and ethylene generate concomitantly. Accordingly two hallmarks of resistance are activated, the hypersensitive reaction (HR) or localized cell death in the vicinity of attack and systemic acquired resistance (SAR) in distal parts of the host. In addition, resistant plants are also furnished with a gene-for-gene recognition system for those pathogens which suppress the PAMP-triggered immunity. This reaction is mediated by a receptor-ligand interaction involving host resistance (R) proteins and the pathogenic effector molecules, thereby neutralizing the activity of effectors and known as effector-triggered immunity [7, 9].

Mungbean yellow mosaic India virus (MYMIV), classified within the *Geminiviridae*, draws particular attention as it infects several legume species inciting the yellow mosaic disease (YMD). YMD inflicts heavy yield reduction at high infection ratesin all blackgram (*V*. *mungo*) producing areas but is exceptionally devastating in the South-East Asian countries [10]. The disease forms large-scale epidemic under optimal environmental conditions imposing 20–80% loss in harvest. Successful pathogenesis accompanies a high degree of morphological and physiological alterations resulting in an appearance of bright yellow chloretic spots on infected leaves.

Enhancement in genetic resistance is an aim in any crop improvement programme that can be accomplished by acquiring knowledge on the genetic basis of resistance. Most of the previous studies focused mainly on inheritance pattern of MYMIV-resistance [11, 12] and their introgression to achieve durable resistance in *V*. *mungo* [10]. Possible involvement of the R-gene in *V*. *mungo*defence has also been investigated focusing on the molecular basis of effector coat-protein recognition of MYMIV [13]. Nevertheless, mounting durable resistance not only necessitates introgression of the R-gene but also entail inclusion of other sources of horizontal resistance to broaden genetic competence to restrict the pathogen invasion [14, 15]. Such non-race-specific resistance is governed by several interacting genes that co-act in concert limiting the pathogen at the site of infection. Use of this strategic control based on horizontal resistance relies on the prior knowledge of the genes associated with the resistance mechanism. However, there was no existing information available which could enlighten about the immune response of *V*. *mungo* in general.

Previously we had undertaken a time course proteomic study to identify the differentially expressed proteins during MYMIV-*V*. *mungo* interactions [16]. The study demonstrated biochemical and proteomic alterations associated with YMD to comprehend protein modulations that commence upon viral infection in resistant and susceptible backgrounds. However, the study was conducted at a later stage of infection (3, 7 and 14 dpi) hence it is difficult to speculate the early events that govern MYMIV-resistance or susceptibility. As the molecular controls of such complex interactions are largely unknown, one approach should be the exploration of post infection transcriptional modulations occurring in the host system. On this backdrop, the technique of suppression subtractive hybridization (SSH) was adopted during this investigation to compare the patterns of infection responsive reactions during the early phases of plant pathogen interactions. The present study illustrates transcriptional reprogramming as an indicator of host immune responses, demonstrating a cascade of signaling events that allow rapid switching to a defence mode deciding the phenotype upon MYMIV inoculation.

## Material and Methods

### Plant material and growth conditions

MYMIV-resistant *Vigna mungo* VMR84 and susceptible cultivar T9 were selected based on their contrasting response to MYMIV. T9 is a high yielding cultivar of *V*. *mungo*, characterized by its wider adaptability and highly sensitivity to YMD [10]. VMR84 is a MYMIV-resistant recombinant inbred line, genetically related to T9 having superior yield performance [17]. Seeds after disinfection (0.1% HgCl_2_ for 5 mins and thorough washing in distilled water) were sown in sterile Soilrite soil mix (mixture of peat, vermiculite and perlite) and grown under glasshouse conditions. Growth conditions were adjusted to 25±1°C with 16/8 h light/dark photoperiod (light intensity of 500 μmol m^-2^ s^-1^) and 80% relative humidity. Roughly after 21 days of luxurious growth (until the first trifoliate leaf expanded completely), young plants were subjected to MYMIV-stress treatments.

### Pathogen inoculation procedures and evaluation of MYMIV accumulation

To impart MYMIV-stress, *Bemisia tabaci* (white fly) populations were reared on susceptible *V*. *mungo* plants maintained within insect proof cages in the insectory facility at the Madhyamgram Experimental Farm (MEF), Bose Institute (BI), Kolkata, India (22°4′ N and 88°27′ E; altitude 9 m). Approximately, 25–30 adult flies were confined in each glass trappers and allowed for an acquisition access period of 24 h on symptomatic leaves of naturally infected plants to ascertain their viruliferous nature. Subsequently, the trappers along with viruliferous flies, were attached to the first trifoliate leaf of healthy *V*. *mungo* plants and allowed for 24 h inoculation access period. During this period virus particles were transmitted from the guts of viruliferous flies to the phloem cells of the infected plants. First trifoliate leaves of both the genotypes were mock inoculated with aviruliferous vectors and were kept in separate glass-cages under identical conditions. Pathogen proliferation in inoculated leaf samples were assessed by PCR amplification of a 575 bp DNA fragment (Accession no. HQ221570) encoding a part of the MYMIV coat protein and later confirmed visually by the appearance of yellow mosaic symptoms on the inoculated leaves of susceptible genotype. Samples were harvested at 3, 6, 9, 12, 18, 24, 36 and 48 hours post inoculation (hpi) and immediately frozen in liquid nitrogen for RNA isolation. In this experiment, biological replicates of three infected and three mock inoculated samples were employed for each time point.

TNA (total nucleic acid) was extracted following modified CTAB method [10]. Relative accumulation of MYMIV was evaluated by qPCR as previously reported by following the method of García-Neria and Rivera-Bustamante [18]. Three biological replicates were carried out to determine the viral titer in the inoculated leaves from the susceptible and resistant host as stated by Boyle et al. [19]. Primers for MYMIV-coat protein (CP) gene (CP-F: 5′-GAAACCTCGGTTTTACCGACTGTATAG-3′ and CP-R: 5′-TTGCATACACAGGATTTG AGGCATGAG-3′) were used as an indicator of MYMIV-load and the actin gene was used for data normalization.

### RNA isolation, mRNA extraction and cDNA synthesis

Total RNA was isolated from mock inoculated and infected leaf tissues by using the Trizol reagent (Invitrogen, Carlsbad, CA) treated with DNase-I (Sigma-Aldrich, USA) to eliminate traces of genomic DNA and purified using the RNeasy Plant Mini Kit (Qiagen, USA) following manufacturer’s instruction. Integrity of the isolated RNA samples were assessed by agarose gel electrophoresis and purity and quantity of individual samples were determined spectrophotometrically (NanoDrop 1000 Spectrophotometer, Thermo Scientific, USA). Subsequently, an equal amount of total RNA (50 μg from each time point) were pooled for each sample and used as a starting material for mRNA extraction. mRNA was purified from the pooled total RNA sampl**e**s using the NucleoTrap mRNA Mini kit (Macherey-Nagel, Germany).

### Construction of SSH library and EST-sequencing

**Fig 1:**
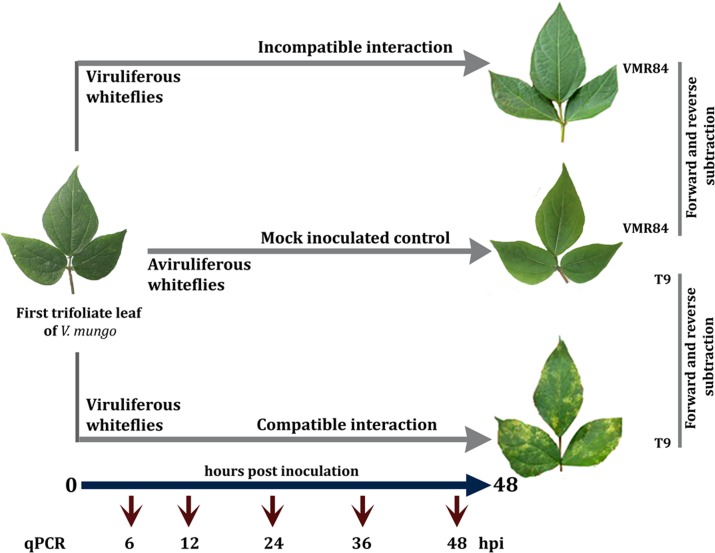
Experimental design for SSH analyses after artificially inoculating resistant (VMR84) and susceptible (T9) *V*. *mungo* genotypes with MYMIV. cDNAs of virus inoculated plants were subtracted in both forward and reverse directions against mock inoculated samples infected with aviruliferous whiteflies. RNA was sampled in replicates at 3, 6, 9, 12, 18, 24, 36 and 48 hpi. Leaf tissues were also sampled for qPCR experiments at the same sampling periods excluding 9 and 18 hpi.

Experiments were conducted using the experimental design as outlined in Fig 1. to identify genes differentially expressed during incompatible and compatible reactions. Leaves being the feeding sites of whiteflies are considered as the primary site for MYMIV perception, where a signaling cascade initiates expression of genes in response to the recognition of foreign intruder. Two *V*. *mungo* cultivars, T9 and VMR84 were selected on the basis of their contrasting responses to MYMIV for this purpose. Both the genotypes were artificially challenged with MYMIV and subtracted in both directions from the respective mock controls, generating forward and reverse SSH libraries for each genotype following the method given below.

Pooled mRNA from MYMIV/mock infected T9 and VMR84 were reverse transcribed to double-stranded cDNA using the SMART PCR cDNA synthesis kit (Clontech, USA) as per manufacturer’s protocol. Obtained cDNA libraries were then subtracted using the PCR-Select cDNA subtraction kit (Clontech, USA) to compare the transcript samples of T9 and VMR84. Consequently four libraries have been generated by reciprocal cDNA subtractions i.e. in both forward and reverse directions. The forward and reverse libraries were obtained by subtracting mock inoculated controls (driver cDNA) from the MYMIV-inoculated samples (tester) and *vice versa*, respectively following manufacturer’s instructions. All PCR amplification reactions were carried out using the Advantage 2 polymerase mix (Clontech, USA). The subtracted cDNA sequences were non-directionally ligated into pGEM-T Easy vector (Promega, USA) and transformed into competent *E*. *coli* DH5α cells. Selection of the transformed clones was based on blue white screening. The transformed white colonies were picked up from an initial Luria agar plate [containing Ampicillin (50 μg/ml), X-gal (20 μg/μl) and IPTG (0.1 mM)] and streaked individually in another culture plate and grown overnight at 37°C. After an initial screening through colony PCR, plasmids from the positive clones were recovered using the PlasmidMinikit (Qiagen). Single-pass Sanger sequencing was performed using the ABI prism 3100 automated DNA sequencers using 25–50 ng of plasmid DNA template and the universal sequencing primers T7 and SP6.

### Sequence processing and EST annotation

EST sequences were trimmed from both sides to remove vector contaminations and adapter sequences using web based application NCBI-vecscreen (http://www.ncbi.nlm.nih.gov/VecScreen/VecScreen.html). ESTs from each library were assembled into contigs using the CAP3 assembly programme (http://pbil.univ-lyon1.fr/cap3.php) following the default parameters. Non-redundant sequences which are greater than 100 bp were only included to produce a differentially expressed unigene dataset.

EST sequences were annotated by homology comparison against the non-redundant NCBI databases using the BLASTn and BLASTx algorithm to assign putative function [20]. Similarity to an annotated sequence was considered to be significant having an E-value less than 1 × 10^–5^. Sequences were also compared using TBLASTx of Dana Farber cancer institute (DFCI) plant gene indices with EST databases of legume species including *Medicago*, *Glycine*, *Lotus*, and *Phaseous* and other model species such as *Arabidopsis*, Riceand *Populus*.

Functional categorization of the ESTs was manually done according to the functional catalogue (FunCat) of the Munich Information Center for Protein Sequences (MIPS). EST sequences obtained were submitted in the EST database (dbEST) of the NCBI (http://www.ncbi.nlm.nih.gov/dbEST/) in compliance with the GenBank guidelines.

### qPCR analysis of selected genes

Real-Time qPCR was conducted to compare the expression pattern of some selected genes during compatible and incompatible interactions. Total RNA was isolated in three independent biological replicates from leaves of MYMIV- and mock-inoculated plants at 6, 12, 24, 36 and 48 hpi. DNase-treated total RNA was reverse transcribed to first strand cDNA using the RevertAid first strand cDNA synthesis kit (Fermentas, Canada) following manufacturer’s instructions. Gene-specific primers for 15 differentially regulated transcripts were custom designed using Primer3 Plus having a GC content of 55–60%, a Tm >50°C, primer length ranging from 18–22 nucleotides and an expected amplicon size of 150–250 bp (S1 Table). Each 20-μl reaction comprised of 0.2 μm forward and reverse primer and cDNA synthesized from 50 ng of total RNA. qPCR reactions were carried out with SYBR Advantage qPCR Premix (2X) (Clontech, USA) in a BioRad iQ5 quantitative real time PCR system (Bio Rad, USA) under the following conditions: an initial denaturation at 95°C for 30 sec was followed by 40 cycles of 5 sec at 95°C, 30 sec at 60°C. On completion of each run, a dissociation curve analysis was done to check the specificity of the primers by heating the samples from 65° to 95°C in increments of 0.5°C, each lasting for 5 s. All the reactions were carried out in triplicates including three non-template controls. The obtained threshold (Ct) values were normalized against the reference gene actin [21] and relative fold changes were calculated by the comparative 2^-ΔΔCt^ method [22].

### DAB staining of infected leaves

Hydrogen peroxide accumulation in inoculated *V*. *mungo* leaves were analyzed using 3, 3′-diaminobenzidine tetrahydrochloride (DAB) staining method according to Schraudner et al. [23]. DAB polymerizes in presence of H_2_O_2_ and generates brown colouration. Fully expanded leaves of both genotypes (VMR84 and T9) were harvested at 0, 12, 24 and 48 h after challenging with MYMIV. Excised leaves were vacuum infiltrated with 1 mg ml^-1^ DAB staining solution, pH 3.8 for 5 min. Vacuum was released gently and the procedure was repeated 2–3 times until the leaves were completely infiltrated with the solution and then kept in a plastic box for 5–6 h under high humidity conditions till reddish brown precipitates were observed. Chlorophyll was removed by heating with 96% (v/v) ethanol at 40°C. DAB stained leaves were then fixed with a solution of 3:1:1 ethanol: lactic acid: glycerol and photographed.

Quantification of H_2_O_2_ from DAB infiltrated leaves was determined according to Kotchoni et al. [24]. After grinding the DAB-stained leaves in liquid nitrogen, 0.2 M HClO_4_ was added and centrifuged for 15 min at 12,000g. DAB activity was determined spectrophotometrically by measuring the absorbance at 450 nm. Obtained results were compared against a standard curve generated for known amounts of H_2_O_2_ in 0.2 M HClO_4_-DAB and expressed in terms of mmol gm^-1^ FW. Experiments were repeated three times on 3 individual plants.

### Lipid peroxidation assay

Lipid peroxidation was spectrophotometrically assayed by quantification of malondialdehyde (MDA), the end product of lipid peroxidation as reported by Cakmak and Horst [25]. Briefly, 100 mg leaf tissue was homogenized and extracted with 20 ml of 0.1% TCA solution and the extract was centrifuged for 10 min at 12000g at 4°C. One ml of recovered supernatant was then incubated with 4 ml of 20% TCA containing 0.5% thiobarbituric acid (TBA) for 30 min at 95°C. The reaction was terminated by placing on ice and centrifuging at 12000g for 10 min. Extent of MDA-TBA complex formation was assayed by measuring the absorbance at 532 nm and subtracting non-specific absorbance at 600 nm. Concentration of MDA (expressed as μ mol g^-1^ FW) was calculated using the extinction coefficient of 155 mM^-1^cm^-1^ using the formula:

$$\mathrm{MDA content}\left(\text{nmol}\right)=\Delta \text{Abs}\left(532-600\right)\text{ nm}/1.55\times 10^{5}$$

### Quantification of Photosynthetic efficiency

After MYMIV- and mock inoculations, three leaves from both resistant and susceptible genotypes were analyzed for their chlorophyll fluorescence parameters. Measurements were performed in young fully expanded leaves and the values were normalized with the foliar area for each trifoliate leaf. After dark adaptation, chl a fluorescence was measured using a portable fluorescence spectrometer Handy PEA (Hansatech, King’s Lynn, Northfolk, UK) according to protocol adopted by Strasser et al [25]. The initial (Fo) and the maximum fluorescence (Fm) were recorded and subsequently the variable fluorescence was calculated (Fv = Fm–Fo). Finally, the Fv/Fm ratio was carried out that correlates with the net quantum yield of PSII. Additionally, an energy pipeline model was generated utilizing the measured parameters with Biolyzer HP 3 (Bioenergetics Laboratory, Switzerland) to compare the energy flow of PSII at different levels between the genotypes in mock inoculations and after challenging with the virus.

## Results

### Accumulation of MYMIV in the two *V*. *mungo* genotypes after challenging with the virus

**Fig 2:**
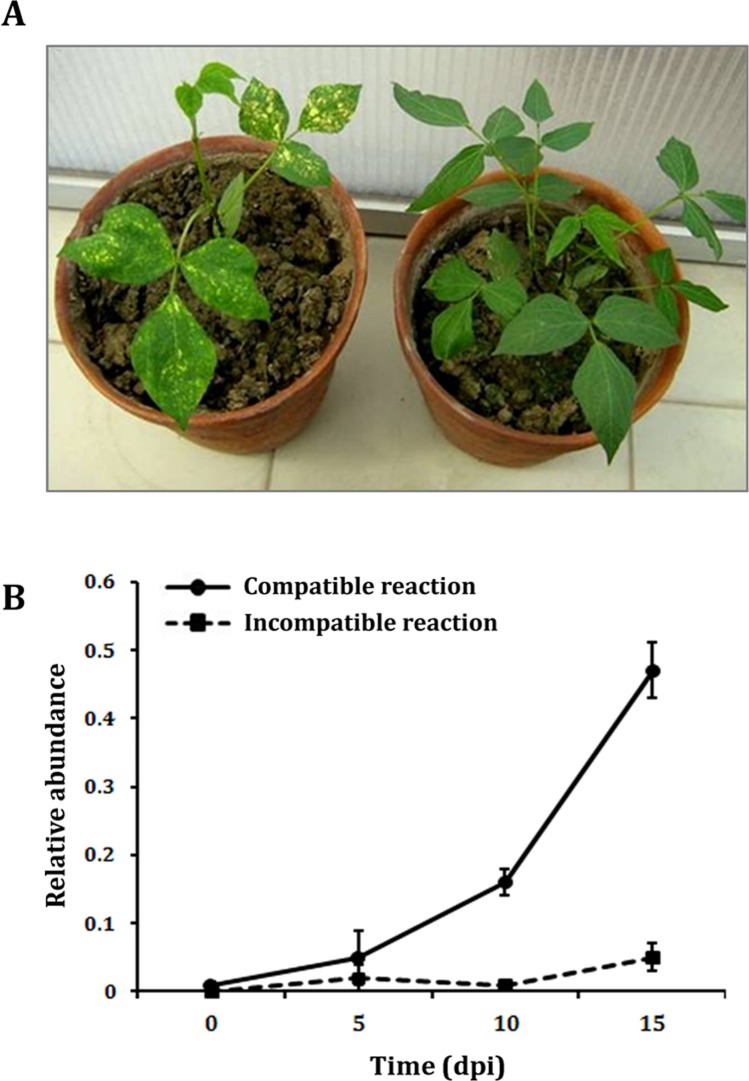
Dynamics of MYMIV infection in *V*. *mungo*. A. Expression of yellow mosaic symptoms in MYMIV-inoculated susceptible (left) and asymptomatic resistant (right) plants at 15 dpi. **B.** Levels of MYMIV-DNA in leaves of inoculated susceptible and resistant plants at different sampling periods. Levels of virus population were monitored *in planta* by qPCR amplification of the CP-DNA from the TNA extracted from inoculated leaf samples at 0, 5, 10 and 15 dpi. The mean values of three biological replicates are presented in the graph. Error bars represent the SDs.

Fate of artificial MYMIV-infection was evaluated based on phenotypic changes along with molecular detection of the MYMIV coat protein (CP) fragment. In the present study, 10 plants from each genotype were challenged with the virus and the phenotypic responses revealed highly resistant nature of VMR84 while T9 demonstrated a susceptible reaction. Development of yellow mosaic pattern on leaves was intimately observed in susceptible plants that started with the appearance of bright yellow specks ultimately coalescing into larger lesions. Although *V*. *mungo* plants were fully symptomatic at 15 dpi, visual symptoms started appearing from 5–7 dpi in the compatible host while establishment of pathogen was severely impaired in resistant VMR84 (Fig 2A). Assuming that the fraction of viral DNA corresponds to the degree of infection, quantitation of MYMIV coat protein (CP) fragment was carried out for a period upto 15 dpi (Fig 2B) to assess the viral titer within inoculated foliar tissue. Low levels of MYMIV-CP were observed in resistant VMR84 plants indicating hindrance in virus proliferation thereby restricting spread of the disease. In contrast, exponential accumulation of MYMIV-CP was reported 5 dpi onwards in susceptible T9. Finally at 15 dpi, a surge in pathogen population reached 1000-fold higher in susceptible T9 than the level calculated in resistant VMR84 genotype correlating with the symptomatic changes in leaf morphology. The results also showed that the mock-inoculated controls remained asymptomatic at all the evaluated time points (data not shown).

### Analysis of transcript sequences

A total of 873 SSH enriched recombinant cDNA clones were identified from four SSH libraries, of which 811 were sequenced, corresponding to 479 and 332 ESTs from resistant and susceptible libraries, respectively. After removal of vector sequences and low quality reads, assembly of ESTs yielded a total of 345 non-redundant tentative consensus (TC) sequences. These includes 205 unigenes derived from the VMR84 library comprising 84 contigs and 121 singletons with an average length of 337 bp. Clustering of 332 T9 ESTs produced 34 contigs and 106 singletons, comprising of 140 unigenes with a mean length of 364 bp. A calculated redundancy of 51.7 and 48.3% in the resistant and susceptible library respectively indicates a high degree of normalization and subtraction efficiency. A summary of the EST statistics have been illustrated in Table 1.

**Table 1:** Overview of EST statistics obtained from the resistant and susceptible genotypes of *V. mungo* upon MYMIV infection.

Description	VMR84	T9
Number of sequenced ESTs	479	332
Number of ESTs after quality control	423	271
Number of unigenes	204	140
Number of contigs	84	34
Number of singletons	120	106
% Redundancy	51.7	48.3
Number of annotated ESTs	170	120
Number of non-annotated ESTs	34	20
Average EST size (bp)	337	364

Transcript analysis revealed a total of 75.1% ESTs induced during incompatible interaction while the majority (57%) was repressed in compatible reaction. SSH data also revealed overlapping expression of 29 ESTs between the compatible and incompatible interactions while the rest 176 and 111 are library-specific sequences of VMR84 and T9 respectively (S1 Fig). All EST sequences were deposited to the GenBank EST database (VMR84: JZ168080- JZ168359; T9: JZ168360- JZ168399).

### Gene annotation and functional classification

Putative functions were assigned to ESTs with various degrees of confidence. Non-redundant ESTs from both resistant and susceptible libraries were searched for homology against the GenBank non-redundant sequence databases using the BLASTX and BLASTN algorithms. It was possible to annotate about 81% of the obtained ESTs with potential functions using the NCBI GenBank database. Rest of the sequences was further compared using the TBLASTX programme of the Dana-Farber Cancer Institute *Vigna unguiculata* (cowpea) Gene Index (DFCI-VuGI, release 8.0) and the TAIR blast programme. This strategy allowed further annotation of 4%, still 53 (15%) sequences did not share homology to any sequences in either database within the set threshold E-value of ≤1 x 10^–5^. Homology search showed that majority of the ESTs (49%) shared homology with soybean sequences (S2 Fig). The percentage sharply decreased to 16.1 in *Medicago* and 9.2 in *Arabidopsis* followed by *Phaseolus* (4.5%) and *Ricinus* (3.8%). These results indicate predominance of legume species in the homology list than other dicot or monocot species. However, lack of *V*. *mungo* in the list signifies the meager amount of sequence information in the database. Collection of novel *V*. *mungo* ESTs resulted from this study therefore serve as a valuable genomic resource for future studies (for complete list of ESTs vide S2 and S3 Tables).

**Fig 3:**
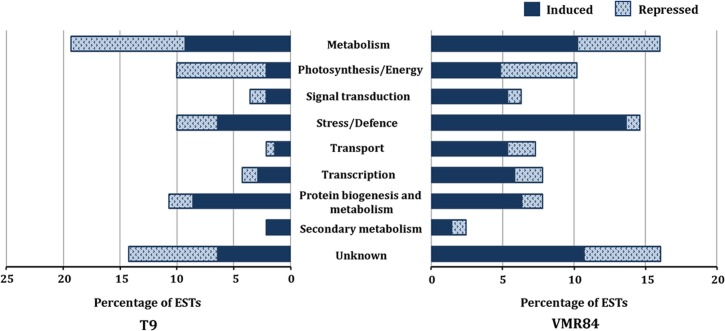
Histograms of the differentially expressed ESTs in VMR84 and T9 after grouping into respective functional categories. ESTs were evaluated for their predicted biological functions through MIPS funcat. The horizontal bars represent the % share of an ontology term determined against the total number of ESTs. Dark and light shaded bars indicate the proportion of induced and repressed ESTs.

ESTs were subsequently categorized into 9 different functional categories based on their putative functions (Fig 3) providing a broad impression on the types of modulated genes in the evaluated genotypes. Majority of ESTs are under the metabolism category that was altered during both compatible (24.8%) and incompatible (18.6%) interactions. ESTs involved in various metabolic pathways like glycolysis (glyceraldehyde 3-phosphate dehydrogenase, fructose bisphosphate aldolase), kreb cycle (malate dehydrogenase, citrate synthase) and photorespiration (glycolate oxidase, serine glyoxylate aminotransferase) showed up regulation in their expression. Several enzymes (cysteine synthase, tryptophan synthase) implicated in amino acid biosynthesis were also overrepresented during incompatible reaction. A significant portion of these ESTs also belongs to the class ‘signal transduction’ showing a higher percentage (6.9%) in the resistant library than in the susceptible libraries (4.4%). Expression of genes coding for protein kinases (MAPK6, STKs), and calcium signaling (calmodulin, calreticulin) were also abundant in VMR84. Modulations of ESTs under “transport” category was higher during incompatible (8%) than compatible (2.7%) interactions, that includes putative potassium transporter, H^+^-ATPases etc. Sizable differences in the number of ESTs were also observed in the category “transcription”. At least 8.5% of SSH clones comprises transcription category in resistant library, while the number declined to 4.4% in the susceptible genotype. The known stress-responsive transcription factors and regulators including WD repeat-containing protein, WRKY, ZF and bHLH are the key components of this category. Although 12% of the ESTs in the susceptible genotype correspond to stress and defence related genes, defence as a category remains overrepresented in resistant VMR84 (16%). Amongst these, several are implicated in pathogen recognition (NBS-LRR, ankyrin, RLKs), oxidative stress (GST, PRX, SOD, TRX), heat shock proteins (HSP90) and other abiotic stresses (CPRD14, CPRD32). Transcripts coding for pathogenesis related proteins such as PR1, PR5 and PR17 having direct role in host immunity were also abundant in the library of incompatible interaction.

Other relevant processes affected during the MYMIV-infestation included “secondary metabolites” and “protein biogenesis and metabolism”. Several transcripts corresponding to the phenylpropanoid pathway were induced in VMR84 reaching 17% of the total. Processes related with “protein biogenesis and metabolism” included 18% of induced genes, but this group also constituted repressed genes, attaining 10% in this case. Protein turnover was also increased as ribosomal proteins, proteases and ubiquitin-related genes are amongst those induced by MYMIV in incompatible interactions.

Contrasting modulation was observed in the photosynthesis/energy class as per their expression is concerned in these two genotypes. While a majority of them showed induced expression in resistant VMR84, abundance of photosynthesis related genes in the repressed list reflects the lower photosynthetic competence accompanying susceptible phenotype of T9.

A major share (17.6%) was assigned to the category “unknown/ unclassified” in the resistant library whereas 17.7% in susceptible library. Efficiency of SSH can be further evidenced by poor representation of the constitutively expressed genes indicating the richness of transcripts associated with defence response. Nonetheless, several transcripts coding forprotein disulfide isomerase, alcohol dehydrogenase etc. that have no apparent defensive roles has also been retrieved.

### Validation of YMD-regulated genes using qPCR

**Fig 4:**
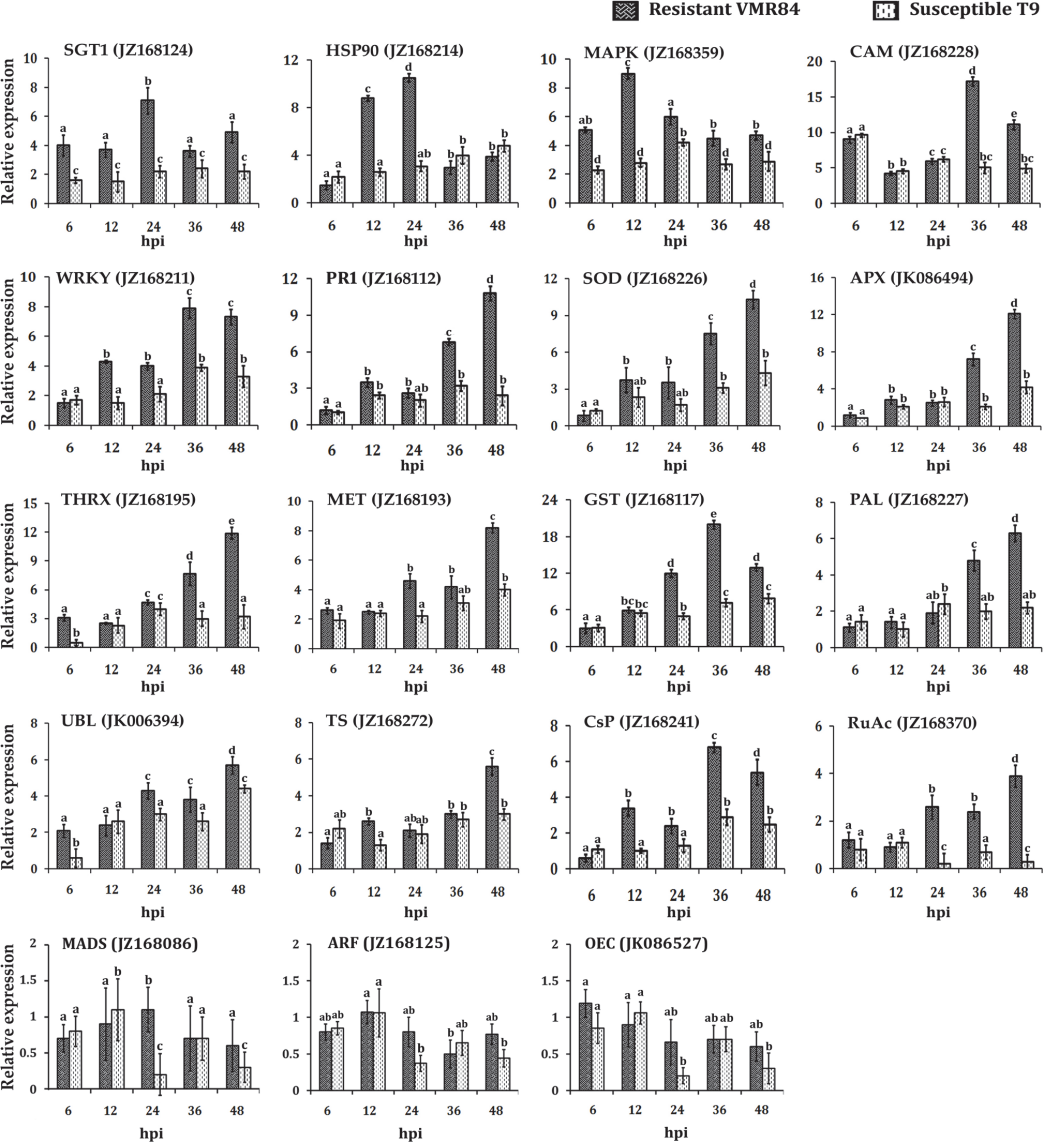
qPCR expression profiles of 16 transcripts that were differentially expressed on challenging compatible (T9: Light bars) and incompatible (VMR84: Dark bars)) plants with MYMIV. Relative expression values were estimated using 2^-Δ(ΔCt)^ method after normalization with actin. Expression analyses were done in five sampling points (6, 12, 24, 36 and 48 hpi) for HSP90 (heat shock protein 90); SGT1; CaM (calmodulin); WRKY transcription factor; GST (glutathione S transferase); APOX (ascorbate peroxidase); Csp (cysteine protease); RuAc (rubisco activase); PAL (phenylalanine ammonia lyase); TRX (thioredoxin); SOD (superoxide dismutase); MET (metallotheionein); PR1 (pathogenesis related protein 1); MAPK (MAP kinase); TS (tryptophan synthase); UBL (ubiquitin ligase) MADS (MADS box protein); ARF (auxin response factor) and OEC (oxygen evolving complex) identified from SSH enriched libraries of either susceptible or resistant blackgram plants. Bars represent mean ± standard deviation; bars followed by different letters indicate significant differences at p ≤ 0.05 according to DMRT.

It is of special interest to explore the expression kinetics of transcripts that clearly discriminate susceptible from resistant reactions. Since SSH does not provide any quantitative estimation of gene expression, therefore qPCR analyses of selected ESTs were undertaken to elucidate the dynamic alterations of gene expression over time. qPCR analyses was carried out at 6, 12, 24, 36 and 48 hpi of different biological replicates (Fig 4). Levels of the accumulated transcripts were normalized against the expression of the *VmACT* gene (JZ078743) that was shown earlier by us as the choice of internal standard for *V*. *mungo* under MYMIV-stress condition [21]. Amplification efficiencies for the primer combinations were observed to be around 2, with a 0.97 correlation in the dilution curve analyses (data not shown). The obtained qPCR expression data of the selected genes are consistent with the results of SSH analyses. Transcripts coding for *SGT1* and *HSP90*, the two functional components of R-protein complex were quantified, maximum expression level noted was 7 and 10 folds at 48 hpi, respectively in VMR84 (Fig 4). SAR indicator gene *PR1* was increased ten times at 48 hpi, triggering the orthodox defence responses against MYMIV.

As anticipated, transcripts coding for ROS homeostasis (*SOD*, *APOX*, *TRX* and *MET*) were all upregulated at 6, 12, 24, 36 and 48 hpi with their highest expression at 48 hpi except for GST which showed a peak of expression (20 fold) at 36 hpi in the incompatible host (Fig 4). In the susceptible host, a late induction of *SOD*, *MET* and *GST* was recorded yet at a much lower magnitude, while levels of *APOX* and *THRX* can hardly be detected at 48 hpi. Trends of expression of these genes indicate further accumulation beyond the observed time points. Transcripts corresponding to *RuAc* lingered under detectable level in infected susceptible plants; however a cumulative accumulation was noted in resistant host. *MAPK* and *CAM* representing the category “signal transduction” was significantly induced early upon inoculation at 12 (9 fold) and 36 hpi (17 fold) respectively. Expression of *TS* and *UBL* showed a modest increase with time in restricting pathogen in resistant host, VMR84. Transcripts corresponding to *PAL* and *WRKY* were also present in considerable amounts in resistant host but were barely noticed in infected susceptible tissues. Additionally three differentially repressed gens were also quantified (*MADS*, *ARF* and *OEC*) that showed repression on MYMIV inoculation than their respective controls in both the genotypes. The overall trend of qPCR expression data corroborates the SSH results and is useful to devise supplementary hypothesis for the possible involvement of the candidate genes in MYMIV-resistance.

### ROS Accumulation in response to MYMIV- inoculation

**Fig 5:**
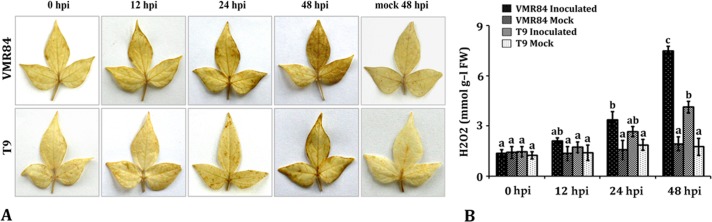
Qualitative (A) and quantitative (B) estimation of H_2_O_2_ in *V*. *mungo* leaves (VMR84 and T9) exposed to MYMIV and mock inoculated plants. DAB staining procedure was used to visualize the *in vivo* accumulation of H_2_O_2_ at 0, 12, 24 and 48 hpi that correlate with the intensity of the brown colour. Bars represent mean ± standard deviation; bars followed by different letters indicate significant differences at p ≤ 0.05 according to DMRT.

Abundance of transcripts in the SSH libraries regulating ROS homeostasis prompted us to detect and quantify endogenous H_2_O_2_ levels by DAB infiltration procedures in pathogen inoculated tissues. Examined leaves showed presence of basal levels of H_2_O_2_ and there is not much difference in colouration in susceptible and resistant host at 0 hpi (Fig 5A and 5B). However, time-course study revealed cumulative accumulation of H_2_O_2_ producing intense colouration in resistant host, VMR84. Quantitative analysis revealed 7.5 mmol g^-l^ FW of H_2_O_2_ at 48 hpi; while detectable level was observed from 12 hpi onwards. In contrast, staining intensity at the site of pathogenic invasion was less prominent in the susceptible reaction exhibiting weaker accumulation. Changes in expression of ROS regulatory transcripts, in both the circumstances, are essential to cope with the amplified ROS levels maintaining a state of balance and initiating systemic redox signaling upon pathogenic invasion.

Membrane damage due to oxidative stress was assayed by quantifying MDA, a consequence produced through reactions involving superoxide radicals generated due to pathogenic infection leading to peroxidation of lipids. Following inoculation, MDA content was found to be similar in mock-inoculated plants of both resistant and susceptible genotypes, while its abundance differed in the MYMIV-inoculated samples. Extent of lipid peroxidation was found to be statistically elevated (35.6 μ mol/gm) at 48 hpi in compatible interaction but were not altered in the previous time points (S3 Fig). This suggests that membrane damage occurred concomitantly along with the appearance of chlorotic symptoms that occurred in the susceptible background upon MYMIV inoculation. In comparison, a less pronounced increase in MDA content (27.6 μmol/gm) was observed at 48 hpi in the resistant genotype.

### Measurement of photosynthetic parameters

The SSH data revealed a gradual attenuation in the magnitude of gene expression that involved in photosynthesis shortly after infection. To confirm whether such transcript modulation is pathogen induced, we measured the fluorescence induction kinetics of chl a molecules in both mock and MYMIV inoculated leaves at 0, 24 and 48 hpi; but no significant decrease in photosynthetic efficiency was observed during the early hours in the resistant host, VMR84 like that of observed reduction in gene expression level. But there was slight decline in the PSII electron transport and partial blockage of the reaction centers in the susceptible host (S4 Fig). While PS II electron transport and other photosynthetic parameters continued to be efficiently shielded from the hostile effects of the pathogen and the parameters tested remained unaffected in VMR84.

## Discussion

The aim of the present study was to gain an insight into the functional and regulatory networks that is operational in *V*. *mungo* during host-pathogen interaction. The previous study hypothesized a plausible mechanism for MYMIV recognition via the gene-for-gene interaction[13], but the question of how *V*. *mungo* activates immune signaling upon pathogen ingression and what are the molecular determinants that determine reaction types still remain elusive. Therefore a comparison between molecular events occurring during compatible and incompatible reactions provides a better handle to understand the host’s response to the pathogen attack. Here we report first-hand information on the immune responses showed by resistant *V*. *mungo* genotype upon MYMIV-infection that facilitated to decipher the molecular mechanisms underlying YMD resistance.

### Transcript remodeling after pathogen ingression independent of basal defence

Successful MYMIV-inoculation to the host plant is manifested by a surge in viral titer that correlates with progressive symptom development in the susceptible host. On the contrary, the resistance machinery seems to be operational from the early hours of pathogen ingress owing to the low copies of detectable coat protein after artificial inoculation. Induced modulations of gene expression are solely due to MYMIV ingress has been confirmed by the judicious subtraction against the mock-inoculated controls. In addition, genetic relatedness of VMR84 with T9 [17] further eliminates the possibility that YMD-resistance is an outcome of constitutive differences in basal expression of defence related genes between these two genotypes. Combining the above facts, the present findings suggest that the observed transcript remodeling is explicitly a pathogen induced phenomenon and not due to the constitutively expressed basal defence of the host.

### R-gene and its component transcripts during incompatible interaction

**Fig 6:**
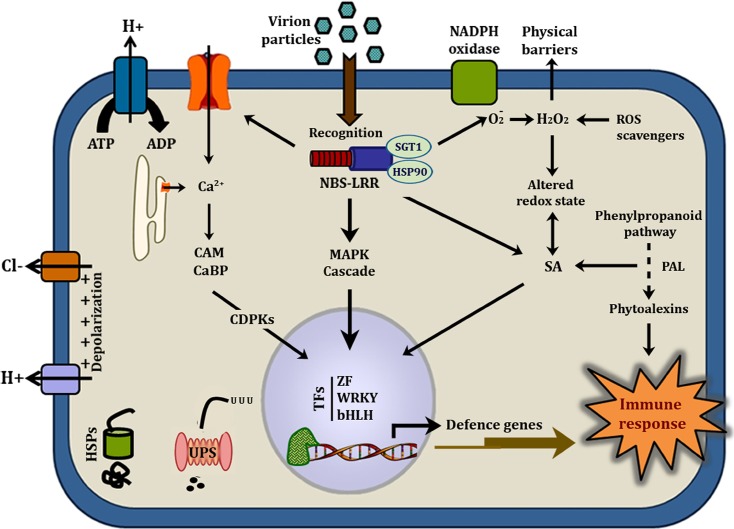
Hypothetical diagram illustrating the molecular mechanism of *V*. *mungo* resistance to MYMIV. The pathway is based on the integrated information collected from the differentially expressed ESTs. NBS LRR: Nucleotide binding site Leucine rich repeat, HSP90: Heat shock protein 90, SGT1: Suppressor of G2 allele of skp1, RAR1: required for Mla12 resistance; MAPK: ROS: Reactive oxygen species; MAP kinases, CAmml: Calmodulin, CaBP: Calcium binding protein, CDPKs: Calcium dependent protein kinases, PAL: Phenylalanine ammonia lyase, SA: Salicylic acid; 26S: UPS: Ubiquitin proteasome system, TFs: Transcription factors, ZF: Zinc finger, bHLH: Basic helix loop helix, WRKY. Bars represent mean ± standard deviation; bars followed by different letters indicate significant differences at p ≤ 0.05 according to DMRT.

Typically, the resistance machinery is recruited upon perception of the invader that activates immune signaling and provides necessary arsenal to arrest pathogen proliferation. In the present study, a comparative transcriptional profiling was conducted to get an insight on the functional and regulatory networks of the early MYMIV stress-responsive genes, based on which a hypothetical model illustrating resistance mechanism operative in *V*. *mungo* against MYMIV has been proposed (Fig 6). Although a broad transcriptional inflection was observed, yet the modulated transcripts do not exhibit any explicit machinery associated with susceptibility. Here compatibility seems to be a consequence of the weak implementation of pathways that are strongly enforced by the resistant host against the intruder. This may be due to the absence of requisite signaling in the susceptible genotype in absence of the candidate resistance gene, *CYR1*, as shown by Maiti et al [13].

Perception of pathogenic attack involves the recognition of pathogen or pathogen derived elicitors (*Avr*) by the host encoded R proteins [9, 26]. Maiti et al. [13] have reported the R-gene mediated recognition of MYMIV-coat protein by the *CYR1* of resistant *V*. *mungo* genotype similar to those demonstrated during host pathogen interactions in *Vitis*-downy mildew [5] and *Glycine*-*Heterodera* [27]. In the present study, one NBS-LRR candidate R-gene was identified which showed induced expression solely in the resistant background. There was also concomitant increase in a tobacco *HSP90* homologue, an integral component of the R-gene complex along with its interacting partner *SGT1* that participates in the assembly, activation and stability of the R-gene products [28]. Hubert et al. [29] and Takahashi et al. [30] showed that silencing of *HSP90* and *SGT1* has led to reduced accumulation of R-proteins compromising the resistance property of the host. Thus, the role of R-gene complex in resistant genotype of *V*. *mungo* is apparent.

### ROS generation vs. scavenging in concurrence with proteomics data

Pathogen induced ROS accumulation at the site of attempted invasion is amongst the earliest cellular concerns associated with host’s responses. We noted a state of balance is maintained between ROS generation and scavenging, as increase in ROS level is detrimental for cell viability [24]. In *V*. *mungo* several redox regulators including PRX, SOD, TRX and GST showed boost in expression level suggesting an imbalance in redox potential. In line with a previous proteomic analysis [16], transcripts of APOX, SOD and GST differed in their induction kinetics with a lower magnitude of expression in the susceptible background. Consistent with the transcript expression data, DAB staining assays provided an additional confirmation showing differential ROS accumulation in these two genotypes. An early state of redox imbalance, especially in the resistant host created the ideal foundation for systemic redox signaling, parallel to the findings reported by Venisse et al. [31] and Manickavelu et al.[4]. Additionally, the greater extent of lipid peroxidation in susceptible background justifies a more pronounced oxidative damage due to the gradual pathogen ingress than in the resistant background.

### Involvement of transcripts in immune signaling

Plants are involved in an intricate interplay of signaling cascade, therefore it can be anticipated that several molecular pathways are in effect during the early hours of pathogen challenge [32, 33]. Defence signaling is well manifested by a rise in Ca^2+^-responders and MAP kinases that participate in the activation of host surveillance against attempted viral invasion. Within the cell, Ca^2+^ levels are maintained precisely through regulation of influx and efflux processes. Effective recognition of the intruder allegedly triggers selective activation of the Ca-channels, elevating its cellular concentration that provides necessary information for signaling [34, 35]. Rise in cytosolic Ca^2+^ level is accompanied by a parallel increase in expression of calreticulins (CRTs) and calmodulins (CAM). A biphasic induction of CAM was witnessed showing an initial burst within few hours in both the genotypes while a more pronounced second phase (16 fold) was observed exclusively in the resistant line. This data corroborates with the proteomic study wherein level of CaM was reported to be much higher during incompatible interaction compared to that of compatible interaction [16]. Besides their role as Ca^2+^ sensor, CRTs are also known to offer hindrance in cell-to-cell trafficking of virion particles restricting pathogen spread in the resistant background [36]. Oscillation in the intracellular Ca^2+^-level stimulates the sensor responders (calcium-binding protein, calcium homeostasis regulator, CHoR1) that activate Ca-dependent protein kinases (CDPKs) [35] and transmit encrypted pathogenic signals further downstream. Although the present findings indicate an early and rapid generation of Ca^2+^ signals, but their frequency, duration and amplitude needs further introspection.

### Role of protein kinases in immune reaction

Compelling evidences suggest the role of protein kinases in bridging pathogen recognition and transcription of responsive genes [37, 38]. Abundant expression of ESTs belongs to the kinase family were noted in the resistant background supporting the above conjecture. Of these, *At*MAPK6 showed 9 fold expression at 12 hpi in the resistant background. The SA induced protein kinases (SIPK), a tobacco ortholog of MAPK6, stimulates N-gene mediated resistance in tobacco [39]. On the other hand silencing of this gene resulted in weak disease resistance in *Arabidopsis* [40]. Notable difference in its abundance in MYMIV-infected VMR84 suggests its participation in SAR. Another fascinating observation is the repression of a MPK4 in VMR84 that negatively regulates SA mediated responses [41, 42]. Interestingly Clarke et al. [43] demonstrated that mpk4 mutants maintained increased level of endogenous SA resulting in the expression of defence related genes and elevated resistance to pathogens. Contrasting expression of these kinases therefore favours SA-pathways suppressing the antagonistic JA/ET-pathways. In an independent proteomics based approach Kundu et al [44] have shown that SA confers tolerance against MYMIV in susceptible *V*. *mungo* plants.

### Dynamics of transcription factors during immune reaction

Transcription factors represent critical hubs in mounting defence reaction by regulating the initial step in gene expression. Here we report four transcription factors ZF, WD40, bHLH and WRKY whose expression were more prominent in incompatible than in compatible reactions. WRKY, a class of plant DNA binding protein recognizes ‘W box’ motifs positioned in the promoters of defence genes induced by biotic stress signals [45]. Here increased abundance of WRKY transcription factors in resistant background at 36 hpi is in conformity with the activation of defence reactions. There are reports demonstrating activation of WRKY transcription factors mediated through MAPK, followed by expression of an array of defence responsive genes to combat pathogen attack [46, 47]. Although interaction between the two partners is beyond the scope of this study, expression profiling suggests WRKY to act downstream of MAPK in the signaling cascade. Even though several members of WRKY have been characterized, only a limited number of ZF, WD40 and bHLH transcription factors have so far been demonstrated in the plant immune system. Overall, the transcription factors play a complementary and/or overlapping role in enhancing expression of downstream components of the defence machinery.

### Ubiquitin proteasome system in determining host-pathogen interaction

Emergence of ubiquitin proteasome system (UPS) influencing plant responses to viruses is somewhat fascinating [48]. Coordinated effort of polyubiquitination of target proteins by ubiquitin ligases and their subsequent degradation by UPS determines the fate of the interaction. However, previous endeavors demonstrating involvement of UPS in restricting pathogen or facilitating infection are somewhat ambiguous [49]. Substantial upregulations of ubiquitin ligase, 26S proteasome subunit beta and RPN7 throughout the course of pathogenesis in VMR84 were noted in this study. Transcript levels of an ubiquitin ligase amplified by 6 fold at 48 hpi in the resistant host, while expressions of the other two genes were not yet determined.

### Differential transcript modulations in resistant and susceptible backgrounds

In general, the MYMIV-elicited transcripts of resistance and susceptible reactions revealed more dissimilarities than commonalities. However, majority of the overlapping responses exhibited a contrasting expression profile with induced levels in VMR84 but suppressed in T9. In particular, several transcripts belonging to stress/ defencecategory demonstrated such disparity confirming the role of paradoxical expression in defining the contrasting reactions. Responses associated with the resistant phenotype include expression of PR genes that are hallmarks of SAR [50]. Three PR gene homologs of Arabidopsis PR1, PR5 and PR17 were identified in *V*. *mungo* that were specifically induced in the resistant background, but not in susceptible. Progressive abundance of PR1 and PR5 suggests SA mediated resistance against MYMIV. Limited expression of the PR genes in T9 may be considered as one of the pathogenic defence suppression strategies. Abundance of PR1 and PR5 proteins during incompatible interaction was also observed through proteomics investigation [16]. Serine/glycine hydroxymethyl transferase, the positive regulator of PR proteins was also found in resistant *V*. *mungo* genotypes indicating a complex interaction in ROS regulation maintaining cellular redox potential. Indeed, susceptibility in T9 is conferred by an inadequate immune response that may be a direct consequence of unsuccessful pathogen recognition resulting in a compatible interaction. While up-regulation in the drought responsive ESTs (CPRD2 and CPRD14) in the resistant genotype reflects crosstalk between biotic and abiotic stress responses. MYMIV is known to depolarize plasma membrane potential due to rapid anion efflux, decreasing water potential of cells and creating drought like consequences [16]. Strong modulation in the expression of CPRD2 and CPRD14 therefore addresses such circumstances eliciting HR in VMR84 as envisaged by Brini and Masmoudi [51].

### Alteration of metabolic and physiological status of infected host

Alteration in the metabolic and physiological status of infected tissue is supported by the existence of a large set of transcripts (18.6% in the resistant and 24.6% in the susceptible background) that functions in host metabolism. Overall repression of this group in T9 correlates with pathogens ability to compete and capitalize on the host’s metabolism. On the contrary, general induction of transcripts of this category in the resistant host suggests a metabolic reprogramming implementing to support the defence mode. Broad analysis of the SSH results indicates specific processes including glycolysis (fructose bisphosphate aldolase), TCA cycle (malate dehydrogenase), starch synthesis (Granule bound starch synthase) and generation of aromatic amino acids (tryptophan synthase) are upregulated in resistant genotype that serves as foundations in the synthesis of antimicrobials. Up-regulation of proteins involved in TCA cycle in the resistant *V*. *mungo* genotype was also perceived through differential proteomic analyses [16] indicating provision for additional energy, an attribute essential for bestowing immune response through the generation of pyruvate and NADPH.

Although biotic stressors are known to repress metabolic processes [5], implications of their induction during early immune phase suggest protection of primary metabolism from foreign intruder. Major shift in the face of protein synthesis is accompanied by induction of rRNA genes encoding large subunit L21, L11 and small subunit proteins S13 S14 and S18. MYMIV aggression also stimulates photorespiratory pathways irrespective of host plant. Early boost in the expression of photorespiratory enzymes (e.g. glycolate oxidase, serine glyoxylate aminotransferase) accounts for intracellular generation of ROS triggered upon pathogen recognition that further increased with disease progression.

### Upsurge of phenylpropanoid pathway at the early hours of virus infection

Among the SSH-enriched cDNA clones, it is important to highlight those involved in the phenylpropanoid pathway guiding the synthesis of physico-chemical barriers and signal molecules implicated in systemic and locally acquired resistance [52]. In this study, up regulation was noted in the expression of PAL, the key regulatory enzyme catalyzing the conversion of phenylalanine to cinnamic acid- an intermediate in the primary and secondary metabolic pathways [53]. A progressive accumulation in the transcript level (6 fold at 48 hpi) suggests its participation in establishing resistance in VMR84. Surveying time points beyond 48 hpi may potentiate in further accumulation, while its levels didn’t reach to comparable amounts within the same time frame in T9 plants. In fact abundance of phenylpropanoid pathway enzymes in the resistant genotype was noted through proteomic investigation at the later stage of MYMIV infection leading to accumulation of SA, phytoalexins, antimicrobials, proline rich cell wall precursor, glycoprotein etc. [16]. All these biomolecules participate in an orchestrated manner in combating virus invasion. However, up-regulation of ubiquitin proteasome system is noted only in early hpi, whereas at later stages of the viral infection carbohydrate flux was redirected towards pentose phosphate pathway to support the cellular energy to a maximum extent [16].

Exogenous application of SA was shown to mimic natural resistance alleviating post infection phenotype in MYMIV-susceptible plants [44]. Increment of SA-induced UDP-glycosyltransferases and FMO1, a hallmark for SA mediated SAR confirms the hypothesis. All these findings taken together strongly support the role of SA in establishing resistance against MYMIV. Additional experiments are required to determine the magnitude and timing of SA accumulation that is concomitant with effective pathogen arrest.

Another trade-off for successful MYMIV infection is the quenching of photosynthesis related genes. Previous effort through a proteomics based approach strongly endorses such modulation accompanied by a drop in chlorophyll content at an advanced stage of infection in susceptible T9 [16]. However, an inductive expression of related transcripts in VMR84 may account for the production of sugar and energy necessary to avert pathogen colonization. Contrasting modulation in expression of rubisco and rubisco activase corroborates the situation with reduced levels in susceptible background at all the evaluated time points. Characteristic mosaics associated with YMD, may be the outcome of viral interference de-prioritizing resources towards hosts defence. Recent works in rice-blast fungus [54] and soybean-*Pseudomonas* interaction [55] identified PS II electron transport as the primary targets of plant viruses. During HR, altered redox state of the cells may interrupt PSII electron transport by damaging its components. FtsH, a chloroplastic zinc dependent metalloprotease, replaces the damaged components restoring PSII function [56, 57]. An increased level of this transcript in VMR84 plants suggests an active PSII repair mechanism while susceptible T9 plants surrender to patho-destruction of PSII. But repression of the photosynthetic genes has no significant effect on host physiology in the early hours, demonstrating that the post infection phenotype due to impaired PSII is strictly restricted to the advanced stages of infection.

Beyond the involvement of reported interactions, participation of non-canonical genes in mediating host defence is of particular interest. This is apparent from the high percentage of ESTs that remained unannotated suggesting a reservoir of uncharacterized genes that are involved in immune response or illustrate novel pathogenicity factors. Annotation of these unknown genes in future will definitely unveil novel mechanisms involved in the pathosystem that remained elusive as yet.

In summary, the present study not only advanced our knowledge base on the regulation of MYMIV-resistance in *V*. *mungo* in genomics perspective; but also highlighted the physiological responses concomitant to mount a fruitful response against MYMIV. Dynamics of gene expression confirmed that the outcome of plant pathogen interaction is a function of gene regulation, showing a clear correspondence between elicitation of immune responsive genes and establishment of host’s response. Comparative transcript analyses representing the early stages in pathogenesis have revealed the participation of both canonical and non-canonical genes during compatible as well as in incompatible reactions. Presumably, resistance is an outcome of extensive transcriptional reprogramming demonstrating an intense interplay of signaling events followed by metabolic changes to implement a defence mode. The present findings depict the coordinate action of SA-responsive pathways, Ca^2+^ signaling, redox imbalance and PR genes in restricting MYMIV, complementing the proteomic data. In contrast, susceptible plants demonstrated a weak implementation of these pathways differing in the induction kinetics and transcript dynamics of stress-responsive genes. Overall repression of the ESTs in the susceptible background can be anticipated as a pathogenic strategy to limit the pathways that are insignificant for the virus life cycle and paving the way for rapid multiplication impeding host defence machinery.

## Conclusion

The present study offered several promising candidate genes providing a valuable genomic resource for future functional analysis addressing mechanisms to translate directly to engineer durable resistance in *V*. *mungo* against the pathogen.

## Supporting Information

S1 Fig Venn diagram representing the number of ESTs in compatible and incompatible reactions. An illustrative diagram showing the number of shared and species-specific ESTs expressed during MYMIV infestation in susceptible and resistant *V*. *mungo* plants.
(JPG)

S2 Fig Bar graph showing the percentage distribution of ESTs across different plant species. X-axis indicates the percentage of ESTs and the Y-axis indicates different plant species indicating relative abundance of ESTs after BLAST analyses.
(JPG)

S3 Fig MYMIV-induced lipid peroxidation in resistant and susceptible *V*. *mungo* genotypes. MDA content (μ mol/ gm FW) of mock and MYMIV-inoculated were quantified at 0, 24 and 48 hpi and represented as a bar diagram. Bars represent mean ± standard deviation; bars followed by different letters indicate significant differences at p ≤ 0.05 according to DMRT.
(TIFF)

S4 Fig Comparative photosynthetic performance showing the phenomenological energy fluxes (per excited cross-section) of resistant and susceptible *V*. *mungo* genotypes. Diagram showing the phenomenological energy fluxes (per excited cross-section) of resistant and susceptible *V*. *mungo* genotypes at 0, 24, 48 and mock 48 dpi after challenging with MYMIV. Absorbed energy: ABS/CSm (absorption maxima per excited cross section), trapped energy: TRo/CSm (trapped energy per cross section), electron transport: ETo/CSm (electron transported per cross section), dissipated energy: DIo/CSm (dissipation maxima per cross section), RC: reaction centres.
(JPG)

S1 Table List of primers used for qPCR analyses. Sequence information and amplicon characteristics of gene-specific primers used for qPCR analyses.
(DOC)

S2 Table Complete list of transcripts showing differential expression in response to MYMIV in resistant background. Two hundred and five sequenced ESTs obtained from resistant genotype are tabulated with EST IDs, annotations (BLASTX similarity), putative function, accession no., size, closest to database match, E-value and expression. The ESTs marked with “#” after EST ID represents the contig sequences while the rest are singletons.
(DOC)

S3 Table Complete list of transcripts showing differential expression in response to MYMIV in susceptible background. One hundred and forty sequenced ESTs obtained from susceptible genotype, T9 are tabulated with EST IDs, annotations (BLASTX similarity), putative function, accession no., size, closest to database match, E-value and expression. The ESTs marked with “#” after EST ID represents the contig sequences while the rest are singletons.
(DOC)
