# Supplementary figures and images for: Transcript Dynamics at Early Stages of Molecular Interactions of MYMIV with Resistant and Susceptible Genotypes of the Leguminous Host, Vigna mungo
Source: PLoS One. 2015 Apr 17;10(4):e0124687. doi: 10.1371/journal.pone.0124687 (PMC4401676; doi:10.1371/journal.pone.0124687)

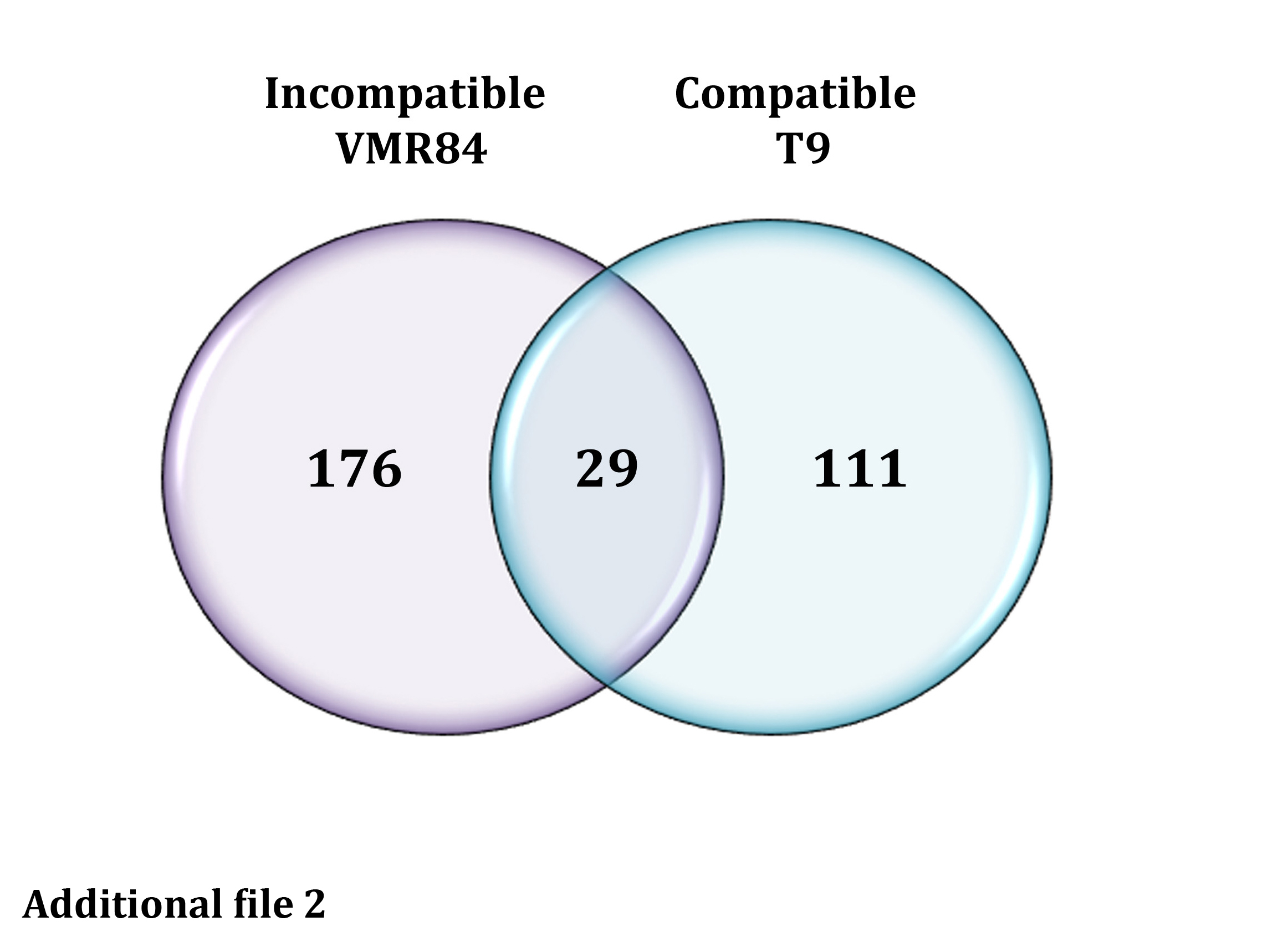

Supplement: S1 Fig — An illustrative diagram showing the number of shared and species-specific ESTs expressed during MYMIV infestation in susceptible and resistant V. mungo plants. (JPG) [file pone.0124687.s001.jpg]

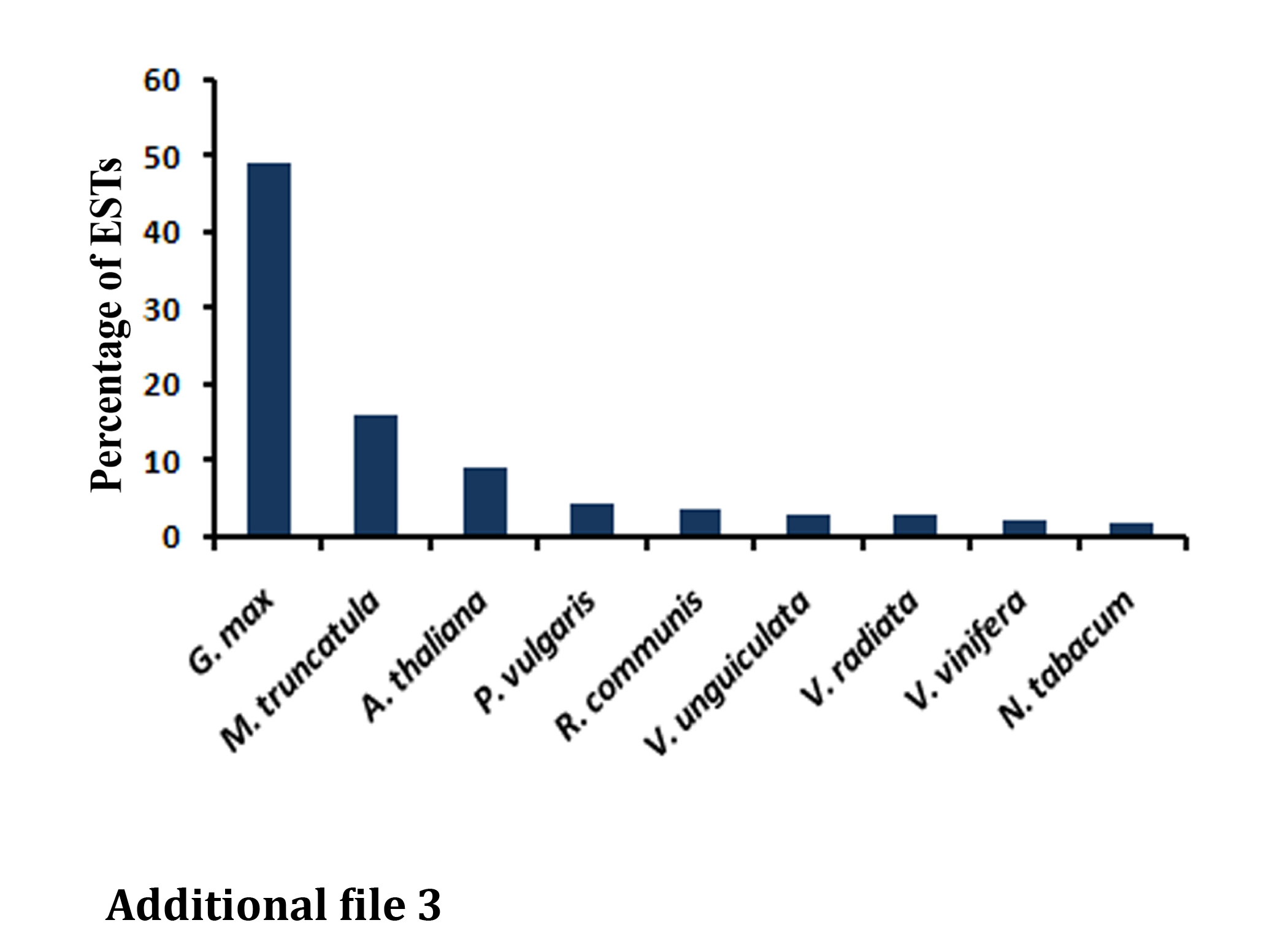

Supplement: S2 Fig — X-axis indicates the percentage of ESTs and the Y-axis indicates different plant species indicating relative abundance of ESTs after BLAST analyses. (JPG) [file pone.0124687.s002.jpg]

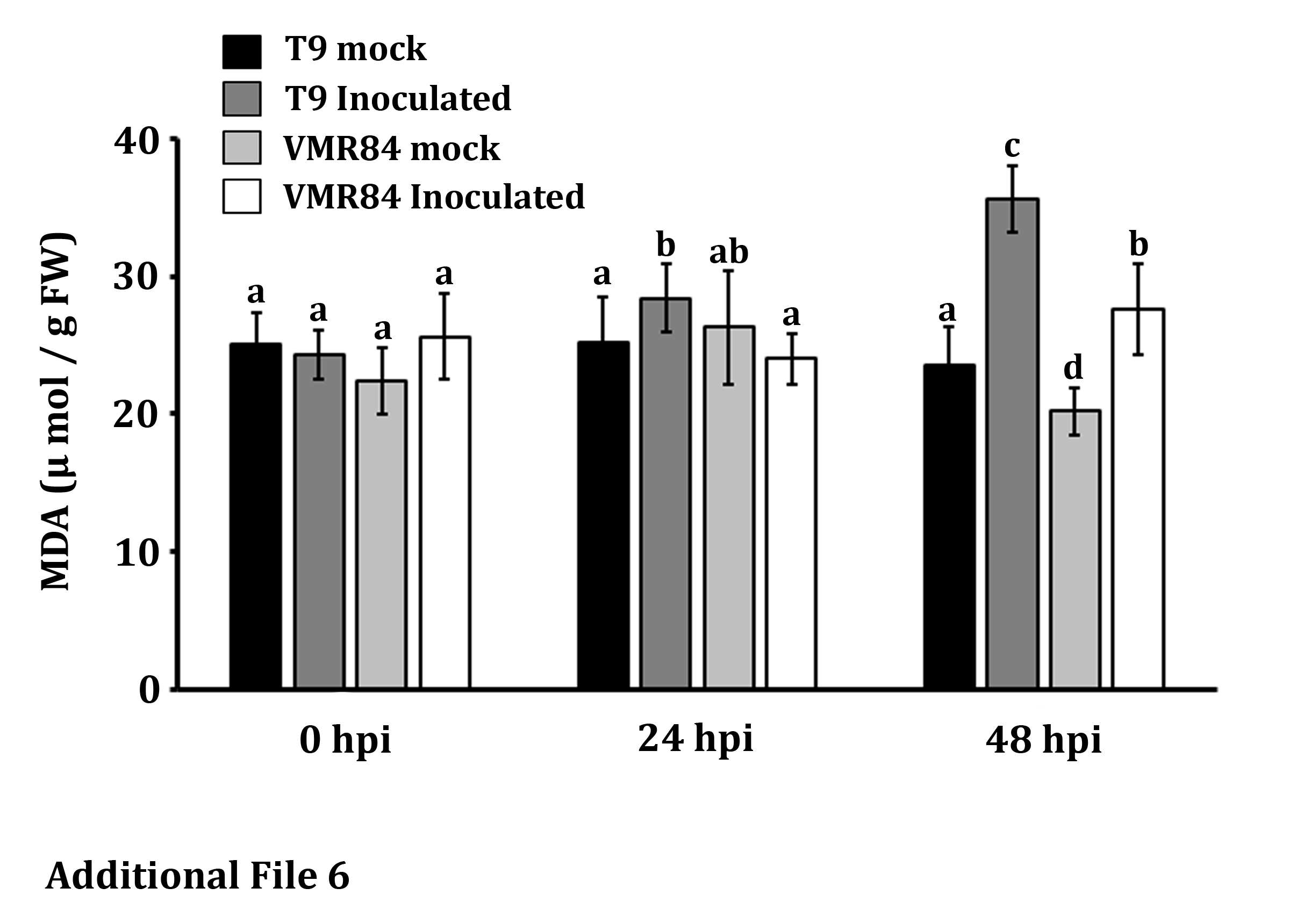

Supplement: S3 Fig — MDA content (μ mol/ gm FW) of mock and MYMIV-inoculated were quantified at 0, 24 and 48 hpi and represented as a bar diagram. Bars represent mean ± standard deviation; bars followed by different letters indicate significant differences at p ≤ 0.05 according to DMRT. (TIFF) [file pone.0124687.s003.tiff]

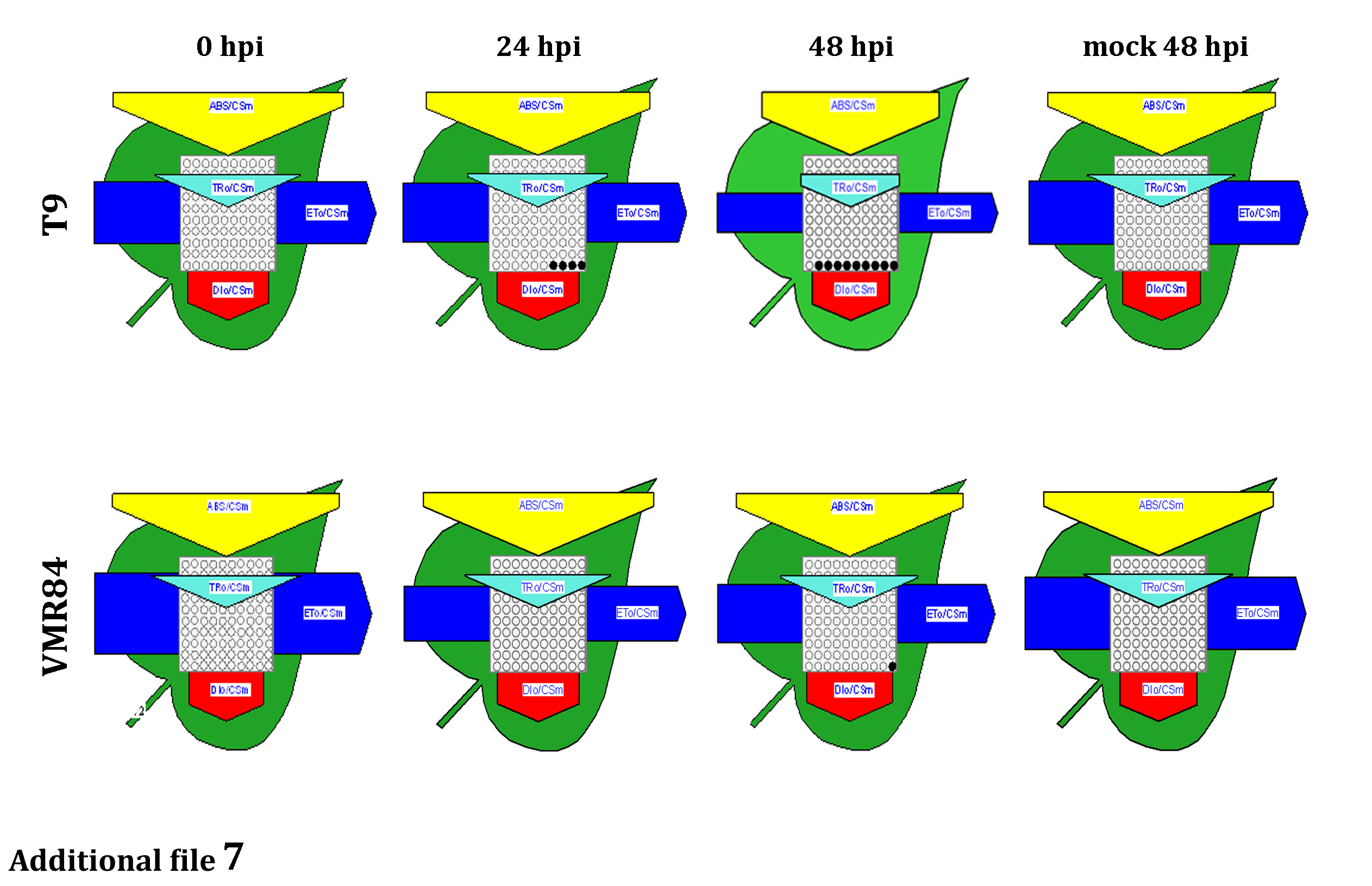

Supplement: S4 Fig — Diagram showing the phenomenological energy fluxes (per excited cross-section) of resistant and susceptible V. mungo genotypes at 0, 24, 48 and mock 48 dpi after challenging with MYMIV. Absorbed energy: ABS/CSm (absorption maxima per excited cross section), trapped energy: TRo/CSm (trapped energy per cross section), electron transport: ETo/CSm (electron transported per cross section), dissipated energy: DIo/CSm (dissipation maxima per cross section), RC: reaction centres. (JPG) [file pone.0124687.s004.jpg]
