# Supplementary material for: Transcript Dynamics at Early Stages of Molecular Interactions of MYMIV with Resistant and Susceptible Genotypes of the Leguminous Host, Vigna mungo
Source: PLoS One. 2015 Apr 17;10(4):e0124687. doi: 10.1371/journal.pone.0124687 (PMC4401676; doi:10.1371/journal.pone.0124687)
Supplement: S1 Table — Sequence information and amplicon characteristics of gene-specific primers used for qPCR analyses. (DOC) [file pone.0124687.s005.doc]

| **Gene name** | **EST ID** | **Primer Sequence** | | **Amplicon length (bp)** |
| --- | --- | --- | --- | --- |
|  |  | **5’-primer** | **3’-primer** |  |
| Heat shock protein 90 (HSP90) | JZ168214 | TTCAAACCCTCCTTGGGACAC | GAATGAAAGCTGGCCAGAAG | 203 |
| SGT1 | JZ168124 | GTTCACCGAAGTCAACAACC | CGGGGTGACTAAAGAAGCTG | 168 |
| Calmodulin (CAM) | JZ168228 | CGAAGAATGCCACAACATGA | CTACTCAGGGCGATTGAAC | 237 |
| WRKY transcription factor (WRKY) | JZ168211 | GATCACGAATCTCTCTCAGG | CGCTGGACCCCTCTAACAC | 238 |
| Pathogenesis related protein 1 (PR1) | JZ168112 | CTGGCAAAGCCAAGAGTGAT | AGCTCTCACAATTATGCAGC | 248 |
| Ascorbate peroxidase (APOX) | JK086494 | CTTGGCACAAAGCCTAGCTC | GAGCAGCCCAATATACTCGC | 164 |
| Rubisco activase (RuAc) | JZ168370 | TGATGGGCGTATGGAGAAGT | TCCCAATAGCATCAACACCA | 186 |
| MAP Kinase (MAPK) | JZ168359 | TCTCTGTCCMCACAAATGAAAAG | CAGAAGATGTGCAGYTTTACTCA | 156 |
| Thioredoxin (TRX) | JZ168195 | GAATCACTCCTCTGCGGTTC | TCCATAGCGGGAGAGATGAG | 168 |
| Tryptophan synthase (TS) | JZ168272 | ACGCTTTGGGTCTGTTTCAT | GCCCATGTTCGTCCTGTAGT | 171 |
| Glutathione S transferase (GST) | JZ168117 | GCTTGAACCTTCCATCAACAA | TCCCGTGCGTTCTCATATT | 151 |
| Cysteine protease (CsP) | JZ168241 | TCCTGCAAATTTGATAAAAGCA | ATGGCATGAGACACCACCA | 190 |
| Metallothionein (MET) | JZ168193 | TTGCAGTTACATGGGTCACAA | TTGACATGAGCTATGCTGAGA | 151 |
| Phenylalanine ammonia lyase (PAL) | JZ168227 | CACTGCATCAGGTGACTTGG | CCAGGGTGGTGCTTCAATTT | 207 |
| Ubiquitin ligase (UBL) | JK006394 | GTTTCGATATGCTTCAGGC | AGAGCTGCCTGAATGAAATG | 139 |
| Superoxide dismutase (SOD) | JZ168226 | CCCTTGTCTGGGGAGGTTAT | GCACATCACACCATCCAAGGT | 209 |
| MADS Box Protein (MADS) | JZ168086 | TTCGAGGCAGGTGACTTTCT | GATCGCGCCTTTCAATTACT | 167 |
| Auxin Response Factor (ARF) | JZ168125 | CTGAGGGGTTTGGCTTGACC | ACCATTACAGACTTCTCAGC | 109 |
| Oxygen evolving complex (OEC) | JK086527 | CAAGAACAGCTGATGGAGATG | ATCAATTCCTTGGACGCAGT | 222 |

**Additional File 1:** Sequences information and amplicon characteristics of gene-specific primers used for qPCR analyses.
