# Supplementary material for: Transcript Dynamics at Early Stages of Molecular Interactions of MYMIV with Resistant and Susceptible Genotypes of the Leguminous Host, Vigna mungo
Source: PLoS One. 2015 Apr 17;10(4):e0124687. doi: 10.1371/journal.pone.0124687 (PMC4401676; doi:10.1371/journal.pone.0124687)
Supplement: S2 Table — Two hundred and five sequenced ESTs obtained from resistant genotype are tabulated with EST IDs, annotations (BLASTX similarity), putative function, accession no., size, closest to database match, E-value and expression. The ESTs marked with “#” after EST ID represents the contig sequences while the rest are singletons. (DOC) [file pone.0124687.s006.doc]

|  |  |  |  |  |  |
| --- | --- | --- | --- | --- | --- |
| **EST ID** | **Accession Number** | **Closest to database match** | **Size (bp)** | **E-value** | **Expression** |
| **Function: Metabolism** | | | | | |
| VMRIL100# | JZ168179 | ACB30382 **|** Granule bound starch synthase Ia [*Vigna radiata* ] | 241 | 1e-29 | Down |
| VMRIL191# | JZ168269 | AAM63663 **|** Peptidyl-prolyl cis-trans isomerase [*Arabidopsis thaliana*] | 351 | 3e-10 | Down |
| VMRIL267# | JZ168346 | NP_001235608 **|** Endo-1,4-beta-mannanase [*Glycine max*] | 303 | 1e-56 | Up |
| VMRIL182 | JZ168260 | ACB30383 **|** Granule bound starch synthase Ib [*Vigna radiata*] | 304 | 2e-23 | Down |
| VMRIL8 # | JZ168087 | AAU14795 **|** Homogentisate phytylprenyltransferase [*Medicago sativa*] | 603 | 2e-79 | Down |
| VMRIL113# | JZ168192 | XP_003524782 **|** Malate dehydrogenase [*Glycine max*] | 559 | 5e-62 | Up |
| VMRIL193 | JZ168272 | NP_001237687 **|** Tryptophan synthase beta subunit [*Glycine max*] | 400 | 9e-60 | Up |
| VMRIL231# | JZ168310 | NP_001237716 **|** Kunitz trypsin inhibitor p20-1-like [*Glycine max*] | 354 | 8e-09 | Up |
| VMRIL3 | JZ168082 | NP_001235267 **|** Cysteine synthase [*Glycine max*] | 311 | 1e-70 | Up |
| VMRIL31 | JZ168110 | AEX09184 **|** Glycolate oxidase [*Gossypium hirsutum*] | 254 | 1e-61 | Up |
| VMRIL137# | JZ168215 | ACP40514 **|** Fructose bisphosphate aldolase [*Medicago sativa*] | 680 | 6e-126 | Down |
| VMRIL164 | JZ168242 | XP_003625999 **|** NifU-like protein [*Medicago truncatula*] | 388 | 1e-17 | Down |
| VMRIL162# | JZ168240 | ACU30051 **|** Branched-chain amino acid aminotransferase [*Glycine max*] | 514 | 5e-78 | Up |
| VMRIL271 | JZ168350 | XP_003529606 **|** Endo-1,3-1,4-beta-D-glucanase [*Glycine max*] | 331 | 2e-48 | Up |
| VMRIL103 | JZ168182 | Q9FS87 **|** Isovaleryl-CoA dehydrogenase [*Solanum tuberosum*] | 259 | 3e-15 | Up |
| VMRIL10 # | JZ168089 | XP_002534080 **|** Histone H2b, putative [*Ricinus communis*] | 524 | 1e-49 | Down |
| VMRIL119 | JZ168198 | XP_003629941 **|** UDP-glucose glucosyltransferase [*Medicago truncatula*] | 200 | 2e-43 | Up |
| VMRIL174 | JZ168252 | ACJ61247 **|** Serine glyoxylate aminotransferase 3 [*Glycine max*] | 317 | 3e-37 | Up |
| VMRIL126 | JZ168205 | XP_003535896 **|** Long chain acyl-CoA synthetase 1 [*Glycine max*] | 385 | 7e-44 | Down |
| VMRIL118 | JZ168197 | ADQ74922 **|** S-adenosylmethionine-dependent methyltransferase [*Jatropha curcas*] | 140 | 2e-22 | Up |
| VMRIL127# | JZ168206 | CAA65540 **|** ADP-glucose pyrophosphorylase [*Pisum sativum*] | 322 | 8e-66 | Up |
| VMRIL143 | JZ168221 | XP_003626495 **|** GDSL esterase/lipase [*Medicago truncatula*] | 478 | 2e-95 | Up |
| VMRIL46 | JZ168125 | XP_003525433 **|** Auxin response factor 2-like [*Glycine max*] | 109 | 2e-07 | Down |
| VMRIL179# | JZ168257 | XP_002280094 **|** Ketol-acid reductoisomerase [*Vitis vinifera*] | 390 | 1e-43 | Down |
| VMRIL111 | JZ168190 | XP_003552681 **|** 2-oxoglutarate dehydrogenase [*Glycine max*] | 260 | 8e-67 | Up |
| VMRIL251# | JZ168330 | XP_003535418 **|** Auxin-induced protein 22B [*Glycine max*] | 413 | 5e-09 | Down |
| VMRIL265# | JZ168344 | XP_003524918 **|** Brassinosteroid-regulated protein BRU1 [*Glycine max*] | 508 | 1e-111 | Up |
| VMRIL110 | JZ168189 | XP_003602786 **|** Protein DEK [*Medicago truncatula*] | 341 | 3e-06 | Up |
| VMRIL157 | JZ168235 | NP_567038 **|** HIS triad family protein 3 [*Arabidopsis thaliana*] | 488 | 4e-78 | Up |
| VMRIL257# | JZ168336 | NP_001030748 **|** Esterase/lipase domain-containing protein [*Arabidopsis thaliana*] | 530 | 5e-09 | Down |
| VMRIL172 | JZ168250 | XP_003602094 **|** Fibrillin precursor-like protein [*Medicago truncatula*] | 258 | 1e-21 | Up |
| VMRIL70# | JZ168149 | CV531018 **|** Auxin-independent growth promoter [*Phaseolus vulgaris* ] | 254 | 4e-08 | Up |
| **Function: Photosynthesis/Energy** | | | | | |
| VMRIL9 | JZ168088 | ABI51594 **|** Chloroplast post-illumination chlorophyll fluorescence increase protein *[Nicotiana tabacum*] | 328 | 4e-46 | Down |
| VMRIL20 | JZ168099 | XP_003546513 **|** Chlorophyll a-b binding protein [*Glycine max*] | 307 | 3e-31 | Up |
| VMRIL15 | JZ168094 | NP_198197 **|** Light-harvesting complex I chlorophyll a/b binding protein 2 [*Arabidopsis thaliana*] | 389 | 3e-45 | Up |
| VMRIL165# | JZ168243 | XP_003609357 **|** Thylakoid lumenal 16.5 kDa protein [*Medicago truncatula*] | 441 | 2e-24 | Down |
| VMRIL181 | JZ168259 | XP_003536189 **|** Chlorophyll a-b binding protein CP26 [*Glycine max*] | 122 | 2e-19 | Down |
| VMRIL108 | JZ168187 | XP_003536189 **|** Chlorophyll a-b binding protein CP26 [*Glycine max*] | 249 | 3e-29 | Down |
| VMRIL109# | JZ168188 | AAD27881 **|** Ribulose-1,5-bisphosphate carboxylase small subunit [*Vigna radiata*] | 416 | 1e-72 | Up |
| VMRIL144 | JZ168222 | ACU14088 **|** Cytochrome b6-f complex Fe-S subunit [*Glycine max*] | 336 | 4e-49 | Up |
| VMRIL186# | JZ168264 | XP_003612185 **|** Ferredoxin-NADP reductase [*Medicago truncatula*] | 348 | 5e-38 | Up |
| VMRIL41 | JZ168120 | NP_568629 **|** NAD(P)H dehydrogenase 18 [*Arabidopsis thaliana*] | 367 | 9e-26 | Up |
| VMRIL156# | JZ168234 | XP_003553172 **|** NAD(P)H-quinone oxidoreductase [*Glycine max*] | 455 | 1e-77 | Down |
| VMRIL40 | JZ168119 | BAF95867 **|** Putative plastid lipid-associated protein [*Vitis vinifera*] | 335 | 3e-50 | Down |
| VMRIL77 | JZ168156 | XP_002532881 **|** DAG protein, chloroplastic[*Ricinus communis*] | 289 | 2e-23 | Down |
| VMRIL115 | JZ168194 | XP_003552331 **|** NADH dehydrogenase [ubiquinone] 1 alpha subunit 1 [*Glycine max*] | 221 | 1e-32 | Up |
| VMRIL21 | JZ168100 | NP_001237135 **|** Glyceraldehyde-3-phosphate dehydrogenase B subunit [*Glycine max*] | 346 | 6e-21 | Down |
| VMRIL154# | JZ168232 | XP_003519141 **|** NADP-dependent glyceraldehyde-3-phosphate dehydrogenase [*Glycine max*] | 466 | 2e-86 | Up |
| VMRIL14 | JZ168093 | XP_003530455 **|** Glutamine-dependent NAD synthetase [*Glycine max*] | 385 | 1e-19 | Up |
| VMRIL131# | JZ168210 | NP_171956 **|** Coenzyme F420 hydrogenase β [*Arabidopsis thaliana*] | 465 | 1e-89 | Down |
| VMRIL35 | JZ168114 | XP_003613199 **|** ATP-citrate synthase [*Medicago truncatula*] | 246 | 2e-33 | Up |
| VMRIL89 # | JZ168168 | BAD93961 **|** Glyceraldehyde 3-phosphate dehydrogenase A subunit [*Arabidopsis thaliana*] | 582 | 9e-69 | Down |
| **Function: Signal transduction** | | | | | |
| VMRIL189 | JZ168267 | XP_002520741 **|** GTP-binding protein, putative [*Ricinus communis*] | 331 | 7e-13 | Up |
| VMRIL180 | JZ168258 | XP_003521228 **|** Receptor-like cytosolic serine/threonine-protein kinase RBK2-like [*Glycine max*] | 376 | 3e-72 | Up |
| VMRIL5 | JZ168084 | XP_003534141 **|** ADP-ribosylation factor GTPase-activating protein [*Glycine max*] | 323 | 2e-56 | Up |
| VMRIL150 | JZ168228 | XP_003602095 **|** Calmodulin [*Medicago truncatula*] | 211 | 1e-20 | Up |
| VMRIL201# | JZ168280 | NP_173259 **|** Calcium-binding protein CML27 [*Arabidopsis thaliana*] | 309 | 2e-20 | Up |
| VMRIL168 | JZ168246 | XP_003613219 **|** ADP-ribosylation factor [*Medicago truncatula*] | 289 | 2e-07 | Up |
| VMRIL204 | JZ168283 | XP_003548645 **|** Mitogen-activated protein kinase homolog MMK2 [*Glycine max*] | 593 | 0.0 | Down |
| VMRIL280# | JZ168359 | NP_181907 **|** Mitogen-activated protein kinase 6 [*Arabidopsis thaliana*] | 404 | 1e-87 | Up |
| VMRIL229# | JZ168308 | XP_003555807 **|** Calreticulin-like [*Glycine max*] | 353 | 5e-17 | Up |
| VMRIL171 | JZ168249 | XP_003538217**|** cAMP response element protein [*Glycine max*] | 198 | 6e-52 | Up |
| VMRIL121 | JZ168200 | ACZ74678 **|** Calcium homeostasis regulator-like protein CHoR1 [*Phaseolus vulgaris*] | 250 | 6e-29 | Up |
| VMRIL58# | JZ168137 | XP_003614178 **|** Receptor-like protein kinase [*Medicago truncatula*] | 367 | 7e-15 | Up |
| **Function: Stress / defence** | | | | | |
| VMRIL194# | JZ168273 | XP_003524899 **|** Stress-associated endoplasmic reticulum protein 2-like [*Glycine max*] | 312 | 1e-37 | Up |
| VMRIL116 | JZ168195 | XP_003545009 **|** Thioredoxin-like protein CDSP32 [*Glycine max*] | 237 | 8e-44 | Up |
| VMRIL202# | JZ168281 | AEZ51831 **|** Pathogenesis-related protein 17 [*Vitis pseudoreticulata*] | 372 | 6e-49 | Up |
| VMRIL33 | JZ168112 | XP_003545772 **|** Pathogenesis-related protein 1- basic [*Glycine max*] | 353 | 1e-30 | Up |
| VMRIL136# | JZ168214 | BAL42333 **|** Heat shock protein 90 [*Nicotiana tabacum*] | 394 | 2e-87 | Up |
| VMRIL148# | JZ168226 | AAF28773 **|** Iron-superoxide dismutase precursor [*Vigna unguiculata*] | 403 | 9e-40 | Up |
| VMRIL75 | JZ168154 | NP_564580 **|** Ankyrin repeat family protein [*Arabidopsis thaliana*] | 268 | 3e-17 | Up |
| VMRIL4# | JZ168083 | ABF13312**|** PR1-like protein, partial [*Phaseolus vulgaris*] | 664 | 4e-51 | Up |
| VMRIL184# | JZ168262 | XP_003592719 **|** Thioredoxin-X [*Medicago truncatula*] | 429 | 9e-59 | Up |
| VMRIL192 | JZ168271 | NP_001235644 **|** TIR-NBS-LRR disease resistance protein [*Glycine max*] | 214 | 1e-18 | Up |
| VMRIL191# | JZ168269 | ABW76504 **|** Pathogenesis-related thaumatin-like protein [*Coffea arabica*] | 373 | 4e-58 | Up |
| VMRIL190 | JZ168268 | ACZ56426 **|** 2-cys peroxiredoxin [*Vigna radiata*] | 311 | 2e-92 | Up |
| VMRIL2# | JZ168081 | XP_003554323 **|** Hypersensitive-induced response protein 1 [*Glycine max*] | 544 | 4e-99 | Up |
| VMRIL42# | JZ168121 | XP_003526679 **|** Pathogen-related protein-like [*Glycine max*] | 220 | 2e-34 | Up |
| VMRIL23 | JZ168102 | XP_003545030 **|** Endoplasmin Hsp90 homolog [*Glycine max*] | 313 | 5e-09 | Up |
| VMRIL101# | JZ168180 | XP_003534053 **|** Peroxidase 47 like [*Glycine max*] | 359 | 7e-22 | Up |
| VMRIL114 | JZ168193 | BAD18374 **|** Type 1 metallothionein [*Vigna radiata*] | 212 | 4e-28 | Up |
| VMRIL160# | JZ168238 | AAP81673 **|** Glutathione peroxidase [*Lotus japonicas*] | 333 | 2e-07 | Up |
| VMRIL203 | JZ168282 | BAA12161 **|** CPRD14 protein [*Vigna unguiculata*] | 177 | 3e-28 | Up |
| VMRIL45 | JZ168124 | XP_003538277 **|** SGT1-like protein (321) [*Glycine max*] | 162 | 3e-46 | Up |
| VMRIL163 | JZ168241 | XP_003533785 **|** Cysteine protease [*Glycine max*] | 361 | 1e-06 | Up |
| VMRIL147 | JZ168225 | BAB33033 **|** CPRD2 protein [*Vigna unguiculata*] | 431 | 2e-48 | Up |
| VMRIL38# | JZ168117 | NP_199030 **|** Glutathione S-transferase [*Arabidopsis thaliana*] | 494 | 6e-20 | Up |
| VMRIL91# | JZ168170 | XP_003529031 **|** Patatin group A-3-like [*Glycine max*] | 530 | 3e-62 | Up |
| VMRIL153# | JZ168231 | XP_003530136 **|** Patatin homolog [*Glycine max*] | 474 | 1e-15 | Up |
| VMRIL197 | JZ168276 | XP_003527650 **|** Snakin-like cysteine rich protein [*Glycine max*] | 175 | 4e-07 | Up |
| VMRIL145 | JZ168223 | XP_002263092 **|** Pathogen-related protein [*Vitis vinifera*] | 220 | 4e-26 | Up |
| VMRIL12# | JZ168091 | XP_002518865 **|** Heat shock protein, putative [*Ricinus communis*] | 509 | 3e-48 | Up |
| **Function: Transport** | | | | | |
| VMRIL199# | JZ168278 | XP_003546567 **|** Probable inositol transporter 2-like [*Glycine max*] | 435 | 2e-79 | Up |
| VMRIL169 | JZ168247 | XP_003529349 **|** Triose phosphate/phosphate translocator [*Glycine max*] | 284 | 2e-10 | Down |
| VMRIL79# | JZ168158 | XP_003531296 **|** Magnesium transporter MRS2-4-like [*Glycine max*] | 444 | 8e-89 | Up |
| VMRIL48# | JZ168127 | XP_003599782 **|** Vacuolar protein sorting-associated protein [*Medicago truncatula*] | 385 | 5e-65 | Down |
| VMRIL166# | JZ168244 | AAB86942 **|** Endoplasmic reticulum HSC70-cognate binding protein precursor [*Glycine max*] | 501 | 3e-48 | Up |
| VMRIL161# | JZ168239 | XP_003546639 **|** ALA-interacting subunit 3-like [*Glycine max*] | 428 | 1e-65 | Down |
| VMRIL19 | JZ168098 | XP_003625625 **|** V-type proton ATPase subunit G [*Medicago truncatula*] | 251 | 1e-12 | Up |
| VMRIL6 | JZ168085 | XP_003606122 **|** Vacuolar sorting protein 4b [*Medicago truncatula*] | 228 | 1e-08 | Down |
| VMRIL44# | JZ168123 | XP_003553202 **|** Non-specific lipid-transfer protein AKCS9 [*Glycine max*] | 341 | 1e-20 | Up |
| VMRIL188 | JZ168266 | XP_003546398 **|** Probable potassium transporter 11 [*Glycine max*] | 368 | 7e-38 | Up |
| VMRIL128 | JZ168207 | CAH58643 **|** Putative peptide transporter [*Plantago major*] | 181 | 2e-12 | Up |
| VMRIL159 | JZ168237 | AAK67706 **|** V-type H+-ATPase subunit A [*Pisum sativum*] | 282 | 3e-59 | Up |
| VMRIL212 | JZ168291 | BAJ25798 **|** Non-specific lipid-transfer protein [*Nicotiana tabacum*] | 350 | 6e-12 | Up |
| VMRIL140 | JZ168218 | FG834341 **|** H^+^ transporting ATPase like protein [*Vigna unguiculata*] | 319 | 5e-12 | Up |
| VMRIL69 | JZ168148 | XP_003540024 **|** Peptide/nitrate transporter At1g22540 [*Glycine max*] | 181 | 7e-17 | Up |
| **Function: Transcription** | | | | | |
| VMRIL139# | JZ168217 | XP_003532448 **|** WD repeat-containing protein 44 [*Glycine max*] | 481 | 1e-42 | Up |
| VMRIL177 | JZ168255 | ACA24493 **|** TFIIS domain-containing protein [*Cucumis sativus*] | 314 | 2e-19 | Up |
| VMRIL132 | JZ168211 | ACG49996 **|** WRKY transcription factor [*Arachis hypogaea*] | 238 | 2e-18 | Up |
| VMRIL122# | JZ168201 | XP_003538310 **|** Homeobox protein BEL1 homolog [*Glycine max*] | 332 | 8e-47 | Up |
| VMRIL196# | JZ168275 | XP_003588527 **|** Zinc finger CONSTANS-like protein [*Medicago truncatula*] | 398 | 9e-15 | Up |
| VMRIL99# | JZ168178 | FF398506 **|** Metallothionein-I gene transcription activator [*Vigna unguiculata*] | 360 | 5e-17 | Up |
| VMRIL87 | JZ168166 | XP_003612909 **|** bHLH transcription factor [*Medicago truncatula*] | 156 | 4e-13 | Up |
| VMRIL195 | JZ168274 | NP_201499 **|** B3 domain-containing protein [*Arabidopsis thaliana*] | 283 | 9e-11 | Down |
| VMRIL209# | JZ168288 | XP_003549984 **|** AP-2 complex subunit mu-like isoform 1 [*Glycine max*] | 321 | 3e-47 | Up |
| VMRIL94# | JZ168173 | XP_003517521 **|** RNA-binding protein 8A-like [*Glycine max*] | 515 | 6e-72 | Down |
| VMRIL81# | JZ168160 | NP_194519 **|** CCT motif family protein [*Arabidopsis thaliana*] | 399 | 5e-14 | Down |
| VMRIL78 | JZ168157 | XP_003620365 **|** Poly(A) polymerase [*Medicago truncatula*] | 331 | 1e-18 | Up |
| VMRIL16 | JZ168095 | XP_003527209 **|** Polyadenylate-binding protein [*Glycine max*] | 166 | 7e-06 | Up |
| VMRIL7# | JZ168086 | XP_003531018 **|** MADS-box protein SVP-like [*Glycine max*] | 299 | 6e-52 | Down |
| VMRIL74 | JZ168153 | FG866334 **|** Zinc finger homeodomain protein SZF-HD [*Glycine max*] | 230 | 7e-17 | Up |
| **Function: Protein biogenesis and metabolism** | | | | | |
| VMRIL36# | JZ168115 | XP_002276634 | 40S ribosomal protein S14-like [*Vitis vinifera*] | 467 | 3e-69 | Up |
| VMRIL82 | JZ168161 | CAA50573 | Translation elongation factor Ts [*Glycine max*] | 154 | 9e-11 | Up |
| VMRIL175 | JZ168253 | CAA09041 | Elongation factor 1-alpha [*Cicer arietinum*] | 186 | 1e-12 | Up |
| VMRIL263# | JZ168342 | AFR23349 | Eukaryotic translation initiation factor 5A [*Arachis hypogaea*] | 381 | 1e-23 | Up |
| VMRIL85 | JZ168164 | XP_003535567 | 40S ribosomal protein S18-like [*Glycine max*] | 141 | 4e-11 | Up |
| VMRIL37 | JZ168116 | XP_003628825 | 50S ribosomal protein L11 [*Medicago truncatula*] | 215 | 1e-16 | Up |
| VMRIL93# | JZ168172 | XP_003638086 | 60S ribosomal protein L21 [*Medicago truncatula*] | 313 | 6e-21 | Up |
| VMRIL258# | JZ168337 | NP_199012 | Metal-dependent protein hydrolase [*Arabidopsis thaliana*] | 434 | 6e-06 | Down |
| VMRIL18# | JZ168097 | XP_003554175 | Zinc-metallopeptidase, peroxisomal-like [*Glycine max*] | 266 | 5e-12 | Down |
| VMRIL17# | JZ168096 | AFR23349 | Eukaryotic translation initiation factor 5A [*Arachis hypogaea*] | 381 | 1e-23 | Up |
| VMRIL187 | JZ168265 | XP_003611248 | 40S ribosomal protein S13 [*Medicago truncatula*] | 341 | 1e-61 | Up |
| VMRIL64# | JZ168143 | XP_003608889 | 26S proteasome subunit RPN7 [*Medicago truncatula*] | 387 | 7e-76 | Up |
| VMRIL57 | JZ168136 | XP_003556404 | ATP-dependent Clp protease proteolytic subunit-related protein 3 [*Glycine max*] | 339 | 2e-19 | Down |
| VMRIL72 | JZ168151 | XP_003527434 | ATP-dependent zinc metalloprotease FTSH [*Glycine max*] | 319 | 2e-73 | Up |
| VMRIL76# | JZ168155 | XP_003549765 | Ribosome biogenesis regulatory protein homolog [*Glycine max*] | 396 | 4e-69 | Down |
| VMRIL49# | JZ168128 | XP_003522675 | Proteasome subunit beta type-6-like [*Glycine max*] | 414 | 3e-51 | Up |
| **Function: Secondary metabolism** | | | | | |
| VMRIL170 | JZ168248 | XP_003590558 |MPBQ/MSBQ methyltransferase [*Medicago truncatula*] | 326 | 4e-65 | Down |
| VMRIL176 | JZ168254 | XP_002513320 | 7-dehydrocholesterol reductase [*Ricinus communis*] | 310 | 3e-69 | Up |
| VMRIL149 # | JZ168227 | AAD45384 | Phenylalanine ammonia-lyase [*Vigna unguiculata*] | 307 | 3e-51 | Up |
| YMVM177# | JK006410 | XP_003538705 | Flavin-containing monooxygenase [*Glycine max*] | 439 | 6e-85 | Up |
| **Function: Unknown** | | | | | |
| VMRIL123# | JZ168202 | XP_003542485 | Unknown [Glycine max] | 380 | 3e-28 | Up |
| VMRIL95 | JZ168174 | AFK42459 | Unknown [*Lotus japonicus*] | 367 | 4e-41 | Down |
| VMRIL71# | JZ168150 | NP_001237977 | Unknown [*Glycine max*] | 317 | 1e-33 | Down |
| VMRIL106# | JZ168185 | ACU19143 | Unknown [*Glycine max*] | 591 | 6e-07 | Down |
| VMRIL173 | JZ168251 | ACU21554 | Unknown [*Glycine max*] | 319 | 6e-18 | Up |
| VMRIL102 | JZ168181 | CBI39617 | Unknown [*Vitis vinifera*] | 240 | 3e-10 | Up |
| VMRIL200 | JZ168279 | AFK35231 | Unknown [*Medicago truncatula*] | 392 | 2e-54 | Down |
| VMRIL80 | JZ168159 | XP_003612435 |Hypothetical protein [*Medicago truncatula*] | 200 | 7e-07 | Down |
| VMRIL13 # | JZ168092 | AFK35231 | Unknown [*Medicago truncatula*] | 384 | 2e-54 | Up |
| VMRIL205 | JZ168284 | NP_001237977 | Uncharacterized protein [*Glycine max*] | 449 | 9e-42 | Up |
| VMRIL83 | JZ168162 | NP_001237706 | Uncharacterized protein [*Glycine max*] | 280 | 1e-41 | Up |
| VMRIL125 | JZ168204 | XP_003545952 | Uncharacterized protein [*Glycine max*] | 236 | 9e-29 | Up |
| VMRIL28# | JZ168107 | XP_003531091 | Uncharacterized protein [*Glycine max*] | 261 | 2e-49 | Down |
| VMRIL92 | JZ168171 | XP_003528570 | Uncharacterized protein [*Glycine max*] | 330 | 2e-61 | Up |
| VMRIL138# | JZ168216 | NP_001238589 | Uncharacterized protein [*Glycine max*] | 448 | 3e-27 | Up |
| VMRIL90# | JZ168169 | XP_003529600 | Uncharacterized protein [*Glycine max*] | 313 | 2e-28 | Down |
| VMRIL97 | JZ168176 | XP_003550079 | Uncharacterized protein [*Glycine max*] | 198 | 1e-14 | Up |
| VMRIL171 | JZ168249 | NP_001239685 | Uncharacterized protein [*Glycine max*] | 409 | 4e-47 | Up |
| VMRIL211 | JZ168290 | NP_001239887 | Uncharacterized protein [*Glycine max*] | 380 | 7e-31 | Down |
| VMRIL120 | JZ168199 | XP_003527108 | Uncharacterized protein [*Glycine max*] | 328 | 7e-22 | Up |
| VMRIL206 | JZ168285 | BAJ95463 | Predicted protein [*Hordeum vulgare*] | 168 | 3e-05 | Up |
| VMRIL117 | JZ168196 | XP_002319476 | Predicted protein [*Populus trichocarpa*] | 281 | 2e-06 | Down |
| VMRIL104# | JZ168183 | ACU19143 | Expressed protein [*Glycine max*] | 580 | 5e-07 | Up |
| VMRIL59 | JZ168138 | No hits found | 133 | – | Up |
| VMRIL60 | JZ168139 | No hits found | 208 | – | Down |
| VMRIL55 | JZ168134 | No hits found | 239 | – | Down |
| VMRIL52# | JZ168131 | No hits found | 147 | – | Up |
| VMRIL67 | JZ168146 | No hits found | 166 | – | Up |
| VMRIL47 | JZ168126 | No hits found | 251 | – | Up |
| VMRIL63 | JZ168142 | No hits found | 146 | – | Down |
