# Supplementary material for: Transcript Dynamics at Early Stages of Molecular Interactions of MYMIV with Resistant and Susceptible Genotypes of the Leguminous Host, Vigna mungo
Source: PLoS One. 2015 Apr 17;10(4):e0124687. doi: 10.1371/journal.pone.0124687 (PMC4401676; doi:10.1371/journal.pone.0124687)
Supplement: S3 Table — One hundred and forty sequenced ESTs obtained from susceptible genotype, T9 are tabulated with EST IDs, annotations (BLASTX similarity), putative function, accession no., size, closest to database match, E-value and expression. The ESTs marked with “#” after EST ID represents the contig sequences while the rest are singletons. (DOC) [file pone.0124687.s007.doc]

| **EST ID** | **Accession Number** | **Closest to database match** | **Size (bp)** | **E-value** | **Expression** |
| --- | --- | --- | --- | --- | --- |
| **Function: Metabolism** | | | | | |
| YMVM156# | JK006389 | XP_003547247 | Beta-glucosidase like [*Glycine max*] | 510 | 3e-64 | Up |
| YMVM164 | JK006397 | BAH89252 | Protein disulfide isomerase L-3b [*Glycine max*] | 379 | 4e-75 | Up |
| YMVM166# | JK006399 | XP_003589533 | Extracellular lipase 2 [*Medicago truncatula*] | 525 | 1e-50 | Up |
| YMVM167 | JK006400 | XP_003613970| CDC50 family protein [*Medicago truncatula*] | 131 | 3e-19 | Up |
| YMVM172 | JK006405 | XP_003551945 | Uncharacterized oxidoreductase [*Glycine max*] | 337 | 3e-70 | Down |
| YMVM180# | JK006413 | XP_003535622 | Rhomboid protein 1-like [*Glycine max*] | 646 | 2e-91 | Up |
| YMVM181 | JK006414 | XP_03519654 | Glucose-1-phosphate adenylyltransferase [*Glycine max*] | 463 | 1e-101 | Down |
| YMVM182 | JK006415 | XP_003607065 | Fructose-bisphosphate aldolase [*Medicago truncatula*] | 680 | 2e-126 | Up |
| YMVM184# | JK006417 | NP_189632 | B12D protein [*Arabidopsis thaliana*] | 510 | 2e-28 | Down |
| YMVM185 | JK006418 | XP_003521319 | Alcohol dehydrogenase class-3 [*Glycine max*] | 517 | 7e-62 | Down |
| YMVM186 | JK006419 | AAF79700 | T1N15.14 [*Arabidopsis thaliana*] | 533 | 4e-20 | Down |
| YMVM190 | JK006423 | XP_003556583 | Peptidyl-prolyl cis-trans isomerase CYP20 [*Glycine max*] | 340 | 2e-18 | Up |
| YMVM192 | JK006425 | XP_003539169 | Glycine cleavage system H protein [*Glycine max*] | 445 | 1e-17 | Up |
| YMVM193# | JK006426 | XP_003590558 | MPBQ/MSBQ methyltransferase [*Medicago truncatula*] | 367 | 4e-65 | Down |
| YMVM196 | JK006429 | AEX09184 | Glycolate oxidase [*Gossypium hirsutum*] | 304 | 1e-61 | Up |
| YMVM203 | JK086486 | XP_002265266 | Cytochrome c [*Vitis vinifera*] | 567 | 6e-11 | Down |
| YMVM208 | JK086491 | XP_003519648 | Alanine aminotransferase 2 isoform [*Glycine max*] | 637 | 1e-144 | Down |
| YMVM209 | JK086492 | XP_002272568 | Gibberellin-regulated protein 9 [*Vitis vinifera*] | 180 | 2e-06 | Down |
| YMVM246 | JK086529 | NP_001235267 | Cysteine synthase [*Glycine max*] | 412 | 1e-70 | Up |
| YMVM247# | JK086530 | XP_003612270 | Senescence-associated protein DIN1 [*Medicago truncatula*] | 420 | 1e-65 | Up |
| YMVM283 | JZ168392 | XP_003630578 |Dual specificity protein phosphatase [*Medicago truncatula*] | 381 | 2e-24 | Up |
| YMVM254 | JZ168363 | XP_003552681 | 2-oxoglutarate dehydrogenase [*Glycine max*] | 333 | 8e-67 | Down |
| YMVM256 | JZ168365 | XP_003556389 | Auxin-responsive protein IAA14 [*Glycine max*] | 320 | 4e-55 | Down |
| YMVM258 | JZ168367 | XP_003591499 | Indole-3-acetic acid-induced protein ARG2 [*Medicago truncatula*] | 126 | 8e-08 | Down |
| YMVM262 | JZ168371 | XP_003527404 | MAK16-like protein [*Glycine max*] | 341 | 8e-05 | Down |
| YMVM271 | JZ168380 | XP_003521298 | Dolichyl-diphospho oligosaccharide-protein glycosyltransferase subunit 2 [*Glycine max*] | 420 | 1e-67 | Up |
| YMVM270# | JZ168379 | NP_001236366 | Granule-bound starch synthase [*Glycine max*] | 336 | 1e-43 | Up |
| **Function: Photosynthesis/Energy** | | | | | |
| YMVM151 | JK006384 | XP_003536189 | Chl a-b binding protein CP26 [*Glycine max*] | 301 | 4e-99 | Down |
| YMVM158 | JK006391 | AAD27881 | Ribulose-1,5-bisphosphate carboxylase small subunit [*Vigna radiata*] | 165 | 6e-29 | Up |
| YMVM171 | JK006404 | XP_003522675| Thylakoid membrane phosphoprotein [*Arabidopsis thaliana*] | 204 | 3e-33 | Down |
| YMVM173# | JK006406 | XP_003552261 | Chlorophyll synthase [*Glycine max*] | 304 | 1e-18 | Down |
| YMVM210 | JK086493 | XP_003519689 | Chlorophyll a-b binding protein 3 [*Glycine max*] | 413 | 1e-48 | Down |
| YMVM191 | JK006424 | XP_003545381 | Chlorophyll a-b binding protein 3, chloroplastic-like isoform 1 [*Glycine max*] | 418 | 5e-65 | Down |
| YMVM214 | JK086497 | XP_003526631 | Photosystem II 22 kDa protein [*Glycine max*] | 512 | 6e-53 | Down |
| YMVM215# | JK086498 | XP_003631913 | Photosystem I reaction center subunit N, chloroplastic [*Vitis vinifera*] | 386 | 2e-74 | Down |
| YMVM218 | JK086501 | ABG36120 | PSI P700 apoprotein A2 [*Nicotiana tabacum*] | 459 | 4e-31 | Down |
| YMVM221 | JK086504 | XP_002265774 | Chloroplast ferredoxin-NADP reductase [*Vitis vinifera*] | 478 | 2e-101 | Down |
| YMVM244 | JK086527 | XP_003551942 | Oxygen-evolving enhancer protein 2 [*Glycine max*] | 300 | 3e-59 | Down |
| YMVM248# | JK086531 | XP_002516464 | NADH-plastoquinone oxidoreductase, putative [*Ricinus communis*] | 418 | 6e-28 | Down |
| YMVM259 | JZ168368 | AAG24884 | Ribulose-1,5-bisphosphate carboxylase small subunit rbcS3 [*Glycine max*] | 171 | 4e-27 | Up |
| YMVM261# | JZ168370 | NP_001240245 | Ribulose bisphosphate carboxylase/oxygenase activase, chloroplastic-like [*Glycine max*] | 705 | 3e-30 | Up |
| **Function: Signal transduction** | | | | | |
| YMVM175# | JK006408 | AFW90518 | SOS2-like protein kinase [*Phaseolus vulgaris*] | 484 | 6e-92 | Up |
| YMVM228 | JK086511 | XP_003547937 | TSL-kinase interacting protein 1[*Glycine max*] | 301 | 1e-17 | Up |
| YMVM238# | JK086521 | NP_198445 | Protein kinase-like protein [*Arabidopsis thaliana*] | 376 | 2e-64 | Down |
| YMVM245 | JK086528 | NP_001235809 | SOS2-like protein kinase [*Glycine max*] | 326 | 2e-07 | Up |
| YMVM268 | JZ168377 | XP_003530955 | Calcium-binding protein Ole e 8 [*Glycine max*] | 211 | 1e-27 | Down |
| **Function: Stress / defence** | | | | | |
| YMVM178 | JK006411 | AAR01523 | Cytosolic class I small heat shock protein [*Nicotiana tabacum*] | 211 | 8e-08 | Up |
| YMVM211 | JK086494 | XP_003606510 | Cytosolic ascorbate peroxidase [*Medicago truncatula*] | 325 | 1e-09 | Up |
| YMVM223 | JK086506 | CAJ43591 | Snakin-like cysteine rich protein [*Phaseolus vulgaris*] | 194 | 6e-13 | Down |
| YMVM225# | JK086508 | XP_003519127 | Peroxiredoxin [*Vigna radiata*] | 409 | 6e-92 | Up |
| YMVM227 | JK086510 | XP_002283030 | Thaumatin-like protein [*Vitis vinifera*] | 521 | 2e-84 | Up |
| YMVM253# | JZ168362 | ADR30064 | Legumin [*Phaseolus vulgaris*] | 553 | 5e-90 | Up |
| YMVM278 | JZ168387 | NP_564580 | Ankyrin repeat family protein [*Arabidopsis thaliana*] | 268 | 4e-21 | Down |
| YMVM290 | JZ168399 | XP_003526679 | Pathogen-related protein-like [*Glycine max*] | 271 | 1e-48 | Down |
| YMVM289 | JZ168398 | BAH01716 | PR-5 protein [*Glycine max*] | 214 | 6e-24  Contd. | Up |
| YMVM286# | JZ168395 | FG832182 | Heavy metal transport/detoxification protein [*Vigna unguiculata*] | 237 | 3e-13 | Down |
| YMVM261 | JZ168370 | XP_00353938 | 70 kDa heat shock-related protein, chloroplastic-like [*Glycine max*] | 173 | 3e-29 | Up |
| YMVM267# | JZ168376 | AAF28773 | [Iron-superoxide dismutase precursor [](http://blast.ncbi.nlm.nih.gov/Blast.cgi#alnHdr_6840824)*Vigna unguiculata*] | 208 | 1e-25 | Up |
| YMVM266 | JZ168375 | XP_003520973 | Metallothionein-like protein 1-like [*Glycine max*] | 172 | 2e-15 | Up |
| YMVM265 | JZ168374 | XP_003630524 | Glutathione transferase [*Medicago truncatula*] | 199 | 1e-29 | Up |
| **Function: Transport** | | | | | |
| YMVM204 | JK086487 | AAY82249| Mitochrondrial voltage-dependent anion-selective channel [*Phaseolus vulgaris*] | 296 | 2e-14 | Up |
| YMVM232 | JK086515 | XP_003532015 | Putative potassium transporter 12 [*Glycine max*] | 277 | 7e-45 | Up |
| YMVM287# | JZ168396 | XP_002529578 | Vacuolar ATP synthase subunit G [*Ricinus communis*] | 251 | 2e-11 | Down |
| **Function: Transcription** | | | | | |
| YMVM207 | JK086490 | XP_003517521 | RNA-binding protein 8A-like [*Glycine max*] | 515 | 6e-72 | Down |
| YMVM233# | JK086516 | XP_003590649 | mRNA-binding protein [*Medicago truncatula*] | 577 | 1e-96 | Up |
| YMVM263 | JZ168372 | XP_002522021 | Zinc finger protein, putative [*Ricinus communis*] | 334 | 6e-57 | Up |
| YMVM259# | JZ168368 | XP_003525326 | WD repeat-containing protein YMR102C-like [*Glycine max*] | 696 | 3e-42 | Up |
| YMVM198 | JK006431 | CAA57551 | Chloroplast RNA binding protein [*Phaseolus vulgaris*] | 452 | 2e-56 | Down |
| **Function: Protein biogenesis and metabolism** | | | | | |
| YMVM153 | JK006386 | XP_003628825 | 50S ribosomal protein L11 [*Medicago truncatula*] | 180 | 6e-16 | Up |
| YMVM161 | JK006394 | ABF13303 | Ubiquitin ligase [*Phaseolus vulgaris*] | 191 | 7e-28 | Up |
| YMVM170 | JK006403 | XP_002531476 | 40S ribosomal protein S7 [*Ricinus communis*] | 246 | 1e-12 | Up |
| YMVM174 | JK006407 | XP_003598463 | 50S ribosomal protein L27 [*Medicago truncatula*] | 296 | 1e-60 | Up |
| YMVM195# | JK006428 | XP_003534318 | ATP-dependent Clp protease proteolytic subunit 4 [*Glycine max*] | 258 | 3e-50 | Down |
| YMVM199 | JK006432 | NP_001154442 | Peptidase M1 family protein [*Arabidopsis thaliana*] | 418 | 1e-20 | Up |
| YMVM275# | JZ168384 | XP_002531476 | 40S ribosomal protein S7 [*Ricinus communis*] | 489 | 5e-95 | Up |
| YMVM217 | JK086500 | XP_003519220 | 60S ribosomal protein L10-like [*Glycine max*] | 264 | 2e-53 | Up |
| YMVM229 | JK086512 | NP_179546 | Proteasome-like protein [*Arabidopsis thaliana*] | 219 | 7e-36 | Up |
| YMVM241# | JK086524 | XP_003611248 | 40S ribosomal protein S13 [*Medicago truncatula*] | 343 | 9e-62 | Up |
| YMVM280 | JZ168389 | AAM65243 | Putative carboxyl-terminal peptidase [*Arabidopsis thaliana*] | 241 | 1e-27 | Up |
| YMVM253# | JZ168362 | XP_003543083 | 60S ribosomal protein L26 [*Glycine max*] | 368 | 3e-17 | Up |
| YMVM157 | JK006390 | XP_003554418 | Threonyl-tRNA synthetase-like [*Glycine max*] | 186 | 1e-35 | Down |
| YMVM255 | JZ168364 | AAM65243 | Carboxyl-terminal peptidase [*Arabidopsis thaliana*] | 241 | 1e-27 | Up |
| YMVM264# | JZ168373 | XP_003533785 | Cysteine proteinase [*Glycine max*] | 433 | 5e-85 | Down |
| **Function: Secondary metabolism** | | | | | |
| YMVM159 | JK006392 | NP_001237637 | Isoflavonereductase homolog 2 [*Glycine max*] | 325 | 1e-22 | Up |
| YMVM236 | JK086519 | XP_003528032 | Ornithine carbamoyltransferase [*Glycine max*] | 481 | 1e-103 | Up |
| **Function: Unknown** | | | | | |
| YMVM264 | JZ168373 | NP_001238589 | Uncharacterized protein precursor [*Glycine max*] | 420 | 3e-27 | Up |
| YMVM160# | JK006393 | XP_003536656 | Uncharacterized protein [*Glycine max*] | 456 | 2e-48 | Down |
| YMVM152 | JK006385 | XP_003554018 | Uncharacterized protein [*Glycine max*] | 515 | 5e-68 | Down |
| YMVM169 | JK006402 | NP_001235818 | Uncharacterized protein [*Glycine max*] | 455 | 8e-36 | Down |
| YMVM176# | JK006409 | XP_003555729 | Uncharacterized protein [*Glycine max*] | 298 | 7e-16 | Up |
| YMVM155# | JK006388 | NP_001242175 | Uncharacterized protein [*Glycine max*] | 321 | 5e-18 | Up |
| YMVM231 | JK086514 | XP_003529600 | Uncharacterized protein [*Glycine max*] | 572 | 2e-28 | Down |
| YMVM275 | JZ168384 | NP_001238325 | Uncharacterized protein [*Glycine max*] | 130 | 3e-13 | Up |
| YMVM260 | JZ168369 | NP_001238413 | Uncharacterized protein [*Glycine max*] | 271 | 9e-39 | Down |
| YMVM187# | JK006420 | XP_003627623 | Hypothetical protein [*Medicago truncatula*] | 331 | 9e-26 | Down |
| YMVM219 | JK086502 | XP_002524516 | Hypothetical protein [*Ricinus communis*] | 380 | 4e-17 | Down |
| YMVM168 | JK006401 | ACJ85976 | Unknown [*Medicago truncatula*] | 268 | 2e-19 | Down |
| YMVM240 | JK086523 | ACU19143 | Unknown [*Glycine max*] | 591 | 6e-07 | Up |
| YMVM224 | JK086507 | ACU19292 | Unknown [*Glycine max*] | 326 | 1e-08 | Up |
| YMVM154 | JK006387 | AFK42543 | Unknown [*Medicago truncatula*] | 284 | 9e-38 | Down |
| YMVM207# | JK086490 | ACU16926 | Unknown [*Glycine max*] | 453 | 2e-69 | Down |
| YMVM194 | JK006427 | XP_002321724 | Predicted protein [*Populus trichocarpa*] | 341 | 1e-06 | Up |
| YMVM237 | JK086520 | XP_002321854 | Predicted protein [*Populus trichocarpa*] | 332 | 6e-25 | Down |
| YMVM220# | JK086503 | No hits found | 270 | – | Up |
| YMVM250 | JK086533 | No hits found | 141 | – | Up |
